# Hemp bark extract attenuates scopolamine-induced cognitive dysfunction through multi-target cholinesterase, MAO-B, and antioxidant mechanisms

**DOI:** 10.1016/j.bbrep.2026.102852

**Published:** 2026-10-28

**Authors:** Hee-Young Heo, Jae Kwon Kim, SeonJu Park, Keum-Soo Song, Satish Balasaheb Nimse, Kooyeon Lee

**Affiliations:** a Research Institute, K-medichem Co., Ltd., Chuncheon, 24341, Republic of Korea; b Metropolitan Seoul Center, Korea Basic Science Institute, Seoul, Republic of Korea; c OBMC Lab, Smart-Healthcare Convergence Major, Hallym University, Chuncheon, 24252, Republic of Korea; d Department of Bio-Health Technology, College of Biomedical Science, Kangwon National University, Chuncheon, 24341, Republic of Korea; e Multidisciplinary Genome Institute, Hallym University, Chuncheon, 24252, Republic of Korea; f Hallym AI-Biohealth R&BD Center, Hallym University, Chuncheon, 24252, Republic of Korea

**Keywords:** Hemp bark extract, Alzheimer's disease, Acetylcholinesterase, Monoamine oxidase-B, Cannabinoids

## Abstract

Cognitive decline associated with Alzheimer's disease is driven by several interconnected mechanisms, including impaired cholinergic signaling, MAO-B-linked oxidative stress, and reduced neuronal antioxidant capacity. In this study, ethanol extracts prepared from hemp root, stem, bark, leaf, and seed were compared to identify hemp-derived materials with multi-target neuroprotective potential. Among the tested extracts, hemp bark extract (HBE) showed the most balanced activity profile, inhibiting acetylcholinesterase, butyrylcholinesterase, and MAO-B with IC_50_ values of 0.56, 0.01, and 0.02 mg/mL, respectively. HBE at 0.2 μg/mL suppressed H_2_O_2_-induced intracellular reactive oxygen species formation and recovered superoxide dismutase activity in SH-SY5Y neuronal cells. In a scopolamine-induced mouse model, oral HBE administration at 100 and 200 mg/kg improved performance in the passive avoidance test and reduced escape latency in the water maze test, indicating protection against scopolamine-induced cognitive deficits. Chemical profiling by ultra-performance liquid chromatography-quadrupole time-of-flight tandem mass spectrometry revealed several cannabinoids in HBE, including cannabidiol, cannabidiolic acid, and cannabichromenic acid. Enzyme assays showed that cannabichromenic acid potently inhibited acetylcholinesterase and butyrylcholinesterase, with IC_50_ values of 1.3 and 6.6 μM, respectively, whereas cannabidiol and cannabidiolic acid were the most active MAO-B inhibitors, with IC_50_ values of 2.3 and 5.3 μM. Docking, GNINA rescoring, molecular dynamics simulations, and MM-GBSA/MM-PBSA analyses further supported stable interactions of these cannabinoids with the target enzymes, particularly cannabidiolic acid with acetylcholinesterase and MAO-B. These findings suggest that HBE protects against scopolamine-induced cognitive dysfunction through coordinated cholinesterase inhibition, MAO-B modulation, and antioxidant defense, with cannabidiol, cannabidiolic acid, and cannabichromenic acid acting as key multi-target constituents.

## Highlights

• HBE attenuates scopolamine-induced cognitive dysfunction in mice.
• HBE inhibits AChE, BuChE, and MAO-B and reduces oxidative stress.
• UPLC-QTOF-MS/MS identified CBD, CBDA, and CBCA in HBE.
• CBCA strongly inhibits AChE and BuChE; CBD/CBDA inhibit MAO-B.
• MD and MM-GBSA support stable cannabinoid binding to enzyme targets.

## Graphical abstract

**Figure:**
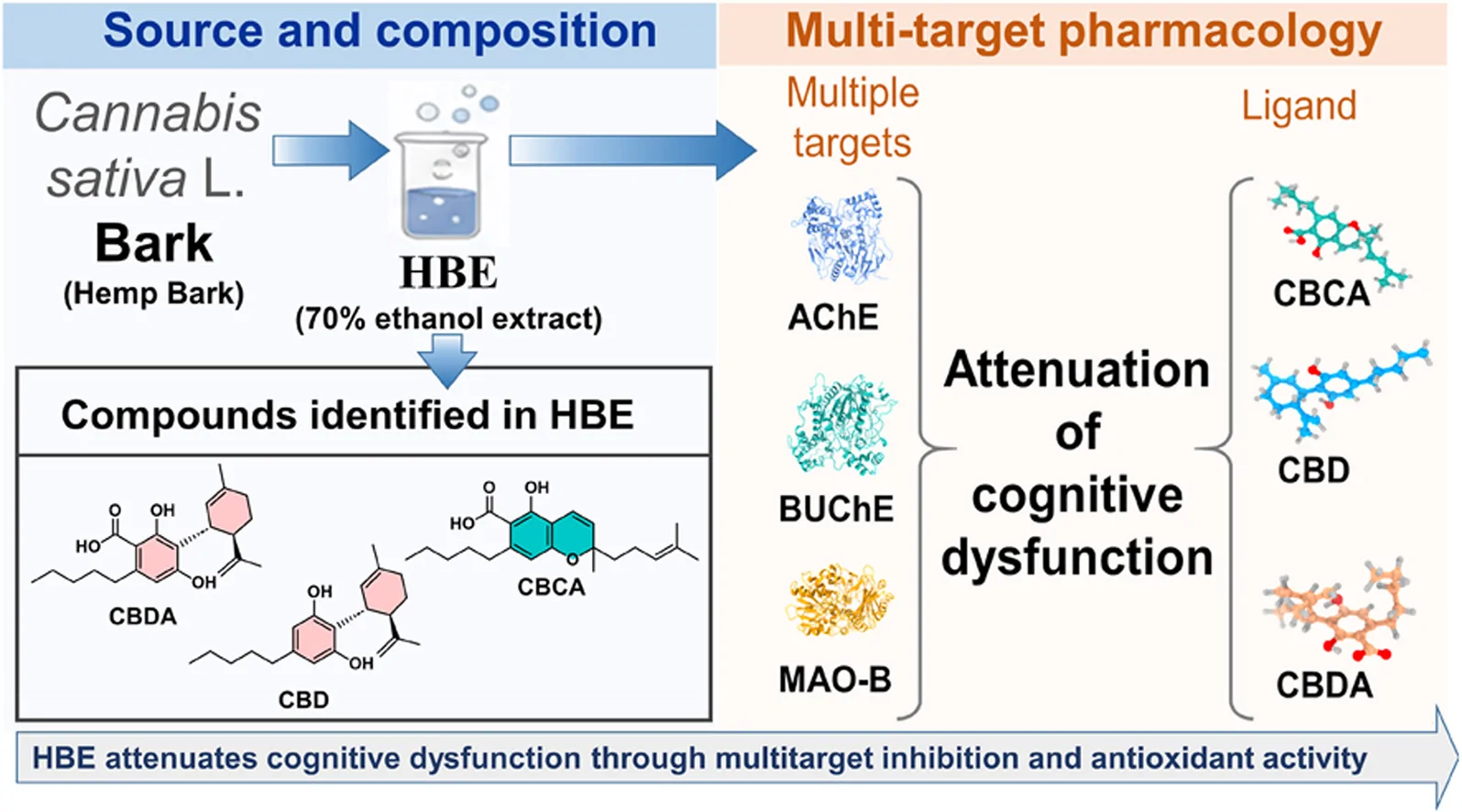

## 1 Introduction

Alzheimer's disease (AD) is a progressive disorder of the central nervous system that gradually disrupts memory, learning capacity, orientation, communication, and higher cognitive functions [1], [2], [3]. With population aging, the burden of AD and related cognitive disorders continues to increase worldwide, creating substantial clinical and socioeconomic challenges [4]. Although considerable progress has been made in defining the molecular basis of AD, effective approaches for preventing or delaying cognitive decline remain inadequate [5], [6]. AD pathology involves several overlapping events, including impaired cholinergic transmission, oxidative injury, mitochondrial dysfunction, neuroinflammation, amyloid-β accumulation, tau-associated abnormalities, and synaptic loss [7], [8], [9], [10]. Accordingly, medicinal plants and structurally diverse natural products that act on multiple pathological pathways are increasingly being explored as sources of candidate modulators for cognitive dysfunction [11].

Cholinergic impairment is strongly associated with memory decline and remains an important therapeutic target in AD and AD-like cognitive dysfunction [12], [13]. Acetylcholine (ACh) regulates learning, attention, memory formation, and synaptic plasticity [14]. During cognitive impairment, loss of cholinergic neurons and reduced ACh-dependent signaling contribute to defective memory processing [15]. AChE terminates cholinergic signaling by hydrolyzing ACh in synaptic regions, while butyrylcholinesterase (BuChE) can also participate in ACh metabolism, particularly under disease-related conditions. Thus, inhibition of cholinesterases continues to be a clinically relevant strategy for symptomatic management of cognitive dysfunction. Dual inhibition of AChE and BuChE may offer broader support of cholinergic neurotransmission than targeting AChE alone [16], [17]. Beyond the cholinergic pathway, MAO-B is also linked to neurodegenerative processes [18], [19], [20].

Elevated monoamine oxidase-B (MAO-B) activity can increase oxidative burden and contribute to oxidative stress-related neuronal damage. Oxidative imbalance is also directly involved in neuronal dysfunction, as excessive ROS production can disturb mitochondrial activity, damage cellular biomolecules, activate inflammatory pathways, and promote neurodegeneration. Antioxidant enzymes, including SOD, are essential for maintaining intracellular redox homeostasis. Therefore, recovery of SOD activity together with suppression of intracellular ROS may reflect a protective response against oxidative insult. Because cholinesterase activity, MAO-B function, and oxidative stress are closely interconnected, compounds capable of influencing these mechanisms simultaneously may be useful for developing multi-target approaches against cognitive impairment. Natural compounds are well suited for this strategy because their chemical diversity can support interactions with multiple biological targets.

*Cannabis sativa* L. is a metabolite-rich medicinal plant that produces cannabinoids, phenolic constituents, flavonoids, terpenoids, lignanamides, and fatty acids [21], [22], [23], [24], [25]. Cannabinoids such as CBD, CBDA, CBC, CBCA, and CBDV have been investigated for anti-inflammatory, antioxidant, neuromodulatory, and cytoprotective activities [26], [27], [28], [29]. However, most pharmacological studies of hemp have focused on flowers [30], leaves [31], [32], seeds [33], or isolated cannabinoids [34], [35], [36]. These and other studies have reported antioxidant, anti-inflammatory, neuromodulatory, cytoprotective, and enzyme-inhibitory activities of hemp-derived constituents [37], [38], [39], [40]. In contrast, hemp bark has received much less attention, despite being an available biomass fraction that may contain bioactive compounds relevant to multi-target modulation of cognitive dysfunction-related pathways.

Here, we compared 70% ethanol extracts prepared from hemp root, stem, bark, leaf, and seed for activities related to cognitive dysfunction. The extracts were evaluated for AChE, BuChE, and MAO-B inhibition, ABTS radical scavenging activity, and protection against H_2_O_2_-induced oxidative stress. The hemp bark extract (HBE) showed the most suitable overall profile, including strong BuChE and MAO-B inhibition, measurable AChE inhibition, reduced intracellular ROS accumulation, and recovery of SOD activity in SH-SY5Y cells. Therefore, HBE was selected for further assessment in a scopolamine-induced short-term cognitive dysfunction mouse model, where it improved performance in passive avoidance and water maze tests. Chemical profiling by ultra-performance liquid chromatography-quadrupole time-of-flight tandem mass spectrometry (UPLC-QTOF-MS/MS) revealed several HBE constituents, including cannabidiol (CBD), cannabidiolic acid (CBDA), and cannabichromenic acid (CBCA), which showed complementary inhibitory effects against AChE, BuChE, and MAO-B. Docking, GNINA rescoring, 100 ns MD simulation, interaction mapping, molecular mechanics generalized Born surface area (MM-GBSA) and molecular mechanics Poisson–Boltzmann surface area (MM-PBSA) analyses further supported stable binding of these cannabinoids within the target enzyme pockets. Overall, this study identifies HBE as a multi-target hemp-derived extract acting through cholinesterase inhibition, MAO-B modulation, and antioxidant defense.

## 2 Materials and methods

### 2.1 Materials and reagents

Hemp plant material was obtained from Nature and Life Company, Hongcheon, Republic of Korea. Before extraction, the plant material was dried under ambient conditions. SH-SY5Y human neuroblastoma cells were obtained from the Korean Cell Line Bank, Seoul, Republic of Korea. Dulbecco's Modified Eagle's Medium (DMEM), fetal bovine serum (FBS), trypsin-EDTA, and penicillin-streptomycin were purchased from Gibco, Gaithersburg, MD, USA, and used for routine cell culture. Acetylcholinesterase (AChE), butyrylcholinesterase (BuChE), butyrylcholine iodide, scopolamine, hydrogen peroxide (H_2_O_2_), 2,2′-azino-bis(3-ethylbenzothiazoline-6-sulfonic acid) (ABTS), 5,5′-dithio-bis-(2-nitrobenzoic acid) (DTNB), kynuramine dihydrobromide, xanthine, and donepezil (DPZ) were obtained from Sigma-Aldrich, Seoul, Republic of Korea. The BCA protein assay kit and protein lysis buffer were purchased from Thermo Fisher Scientific, Seoul, Republic of Korea. WST-1 [2-(4-iodophenyl)-3-(4-nitrophenyl)-5-(2,4-disulfophenyl)-2H-tetrazolium monosodium salt] was obtained from Dojindo Laboratories, Kumamoto, Japan. MAO-B and H_2_DCFDA were purchased from BD Gentest, Woburn, MA, USA, and Invitrogen, Carlsbad, CA, USA, respectively. Xanthine oxidase was supplied by Roche, Burgess Hill, UK.

### 2.2 Preparation of ethanol extracts from hemp parts

The dried hemp material was separated manually into bark, root, stem, leaf, and seed fractions. Each fraction was further dried at 20–30 °C for three days and then ground into powder using an HMF-3250s mixer (Hanil Electric, Republic of Korea). Extraction was performed with 70% ethanol using an ultrasonic-assisted procedure. Briefly, powdered samples were sonicated for three 15 min cycles at 20 kHz using a Sonopuls HD 4200 homogenizer (Bandelin, Germany). The extract suspensions were filtered under vacuum through Advantec No. 1 and No. 2 filter papers (Toyo Roshi Kaisha, Ltd., Tokyo, Japan). The filtrates were concentrated using a Hei-Vap Advantage rotary evaporator (Heidolph, Schwabach, Germany), following a previously reported extraction procedure with minor modification [41]. The concentrated extracts were lyophilized and stored at −80 °C until use.

### 2.3 AChE and BuChE inhibitory assay

The inhibitory effects of the hemp extracts and selected cannabinoids on AChE and BuChE were determined using a modified Ellman colorimetric assay [42]. The assay monitors the generation of 2-nitro-5-thiobenzoate from DTNB after enzymatic hydrolysis of the corresponding thiocholine substrate. Reactions were conducted at 37 °C in 96-well plates with a final volume of 200 μL. Each reaction contained 50 μL of test sample, 50 μL of AChE or BuChE solution prepared in 100 mM phosphate buffer (pH 6.8; final enzyme concentration, 0.02 U/mL), 50 μL of substrate solution, and 50 μL of DTNB solution at a final concentration of 0.3 mg/mL. Absorbance was recorded at 405 nm using a SpectraMax M5 multi-mode microplate reader (Molecular Devices, Sunnyvale, CA, USA). Enzyme inhibition was calculated relative to the inhibitor-free control using the following equation, where A_sample_ and A_control_ represent the absorbance for sample and control solutions, respectively.

$$\text{AChE}\;\left(\text{or}\;\text{BuChE}\right)\;\text{inhibitory}\;\text{activity}\;\left(\%\right)=\left(1-\frac{A_{\text{sample}}}{A_{\text{control}}}\right)\times 100$$

### 2.4 ABTS radical scavenging assay

The free-radical scavenging capacity of each hemp extract was measured using the ABTS radical cation decolorization method with slight modification [43]. ABTS radical cation was prepared by combining 10 mL of 7 mM ABTS solution with 176 μL of 140 mM potassium peroxydisulfate and allowing the mixture to react for 16 h at room temperature in the dark. Before the assay, the radical solution was diluted with absolute methanol until the absorbance at 732 nm reached approximately 0.70. For each measurement, 100 μL of extract solution at the indicated concentration was mixed with 100 μL of diluted ABTS radical solution and incubated for 10 min at room temperature under light-protected conditions. Absorbance was measured at 732 nm using the SpectraMax M5 plate reader. The ABTS radical solution without sample was used as the control. Radical scavenging activity was calculated as follows, where A_sample_ and A_control_ represent the absorbance for sample and control solutions, respectively.

$$\text{ABTS}\;\text{scavenging}\;\text{activity}\;\left(\%\right)=\left(1-\frac{A_{\text{sample}}}{A_{\text{control}}}\right)\times 100$$

### 2.5 MAO-B inhibitory assay

MAO-B inhibition was assessed using a fluorometric kynuramine oxidation assay, adapted from a reported method [44]. Assays were carried out in black 96-well plates. Each well contained 135 μL of 0.1 M potassium phosphate buffer (pH 7.4, adjusted to isotonicity with KCl), 10 μL of kynuramine solution to give a final substrate concentration of 5 μM, and 5 μL of test sample. The extracts were first dissolved in DMSO and diluted with assay buffer before use. After 15 min preincubation at room temperature, 50 μL of MAO-B solution was added to each well to obtain a final enzyme concentration of 10 μg/mL. The reaction was allowed to proceed for 20 min and was then stopped by adding 75 μL of 2 N NaOH. The fluorescent product was measured at an excitation wavelength of 310 nm and an emission wavelength of 380 nm using a microplate reader. MAO-B inhibition was calculated using the fluorescence values of the enzyme control, sample, and blank wells, designated as F_EC_, F_S_, and F_B_, respectively.

$$\text{MAO}-B\;\text{inhibitory}\;\text{activity}\;\left(\%\right)=\frac{\left(F_{\text{EC}}-F_{S}\right)}{\left(F_{\text{EC}}-F_{B}\right)}\times 100$$

### 2.6 Measurement of intracellular ROS in SH-SY5Y cells

Intracellular ROS accumulation was quantified using the H_2_DCFDA fluorescence assay with minor modification [45]. SH-SY5Y cells were maintained in DMEM supplemented with 10% heat-inactivated FBS and antibiotics at 37 °C in a humidified 5% CO_2_ incubator. Cells were seeded in 24-well plates at 2 × 10^4^ cells/well and cultured for 24 h. The medium was then replaced with low-serum medium containing 0.5% FBS. After washing, cells were treated with each hemp extract at 0.2 μg/mL in the presence of 25 μM H_2_DCFDA for 1 h. Oxidative stress was induced by adding 400 μM H_2_O_2_ for 30 min. Oxidation of H_2_DCFDA to fluorescent DCF was measured at excitation/emission wavelengths of 485/545 nm using a microplate reader. Intracellular ROS levels were expressed relative to the control using the following equation, where F_T_, F_C_, and F_B_ represent fluorescence intensities of treated cells, control cells, and background, respectively.

$$\text{Intracellular}\;\text{ROS}\;\text{level}\;\left(\%\;\text{of}\;\text{control}\right)=\frac{\left(F_{T}-F_{B}\right)}{\left(F_{C}-F_{B}\right)}\times 100$$

### 2.7 Determination of SOD activity in SH-SY5Y cells

SOD activity was measured using a xanthine/xanthine oxidase-WST-1 assay, with minor modification of a reported protocol [46]. In this assay, superoxide generated by xanthine oxidase reduces WST-1 to a soluble formazan dye, whereas SOD decreases formazan formation by dismutating superoxide radicals. SH-SY5Y cells were seeded in 6-well plates at 2 × 10^5^ cells/well and incubated for 48 h. The medium was replaced with low-serum medium, and cells were treated with each hemp extract at 0.2 μg/mL in the presence of 200 μM H_2_O_2_ for 24 h. After treatment, cells were washed with ice-cold calcium- and magnesium-free Dulbecco's phosphate-buffered saline and collected by trypsinization. Cell pellets were obtained by centrifugation at 15,000 rpm for 15 min and lysed in 200 μL of RIPA buffer containing protease and phosphatase inhibitors. For the SOD reaction, 6.6 μL of cell lysate was mixed with 186.6 μL of reaction solution containing 216 mM potassium phosphate buffer (pH 7.8), 0.01 mM WST-1, 0.1 mM EDTA, and 55 mM xanthine, followed by addition of 6.6 μL of xanthine oxidase solution (4 U/mL). After incubation at 37 °C for 20 min, absorbance was recorded at 438 nm. SOD activity was normalized to total protein concentration measured using the BCA assay. Relative SOD activity was calculated as follows, where A_sample_ and A_control_ represent the absorbance for sample and control solutions, respectively.

$$\text{SOD}\;\text{activity}\;\left(\%\right)=\frac{\left(A_{\text{sample}}-A_{\text{control}}\right)}{A_{\text{control}}}\times 100$$

### 2.8 *In vivo* experiments

#### 2.8.1 Experimental animals

Seven-week-old male ICR mice were obtained from Hana Biotech Co. (Pyeongtaek, Republic of Korea). The animals were maintained at the Southeast Medi-Chem Institute (Busan, Republic of Korea) under standard laboratory housing conditions and were allowed to acclimatize for one week before the experiment. Food and water were provided ad libitum. All animal procedures were approved by the Institutional Animal Care and Use Committee of the Southeast Medi-Chem Institute (Approval No. SEMI-20-014). After acclimatization, mice were randomly assigned to six groups (n = 5 per group): untreated control, scopolamine alone, scopolamine plus vehicle (VEH), scopolamine plus donepezil (DPZ; 0.75 mg/kg), scopolamine plus HBE 100 mg/kg, and scopolamine plus HBE 200 mg/kg. HBE, DPZ, or vehicle was administered orally once daily for 28 days. Scopolamine was injected intraperitoneally at 1 mg/kg on day 21 before the passive avoidance test and on day 28 before the water maze test to induce short-term cognitive dysfunction. Body weight was recorded weekly during the study period. The experimental schedule is shown in Fig. 3.

#### 2.8.2 Scopolamine-induced short-term cognitive dysfunction mice model

Short-term cognitive impairment was induced by intraperitoneal administration of scopolamine in seven-week-old male ICR mice. Scopolamine was given at 1 mg/kg 30 min before the passive avoidance test on day 21 and the Morris water maze test on day 28 [44]. The passive avoidance test was used to assess contextual fear-associated memory, whereas the Morris water maze test was used to evaluate spatial learning and memory. Body weight, food intake, and water consumption were monitored weekly throughout the experiment.

#### 2.8.3 Administration of hemp bark extract

To assess the preventive effect of HBE, mice received HBE orally at 100 or 200 mg/kg once daily from day 0 to day 28. HBE was prepared in vehicle solution containing 1% DMSO and 1% Tween 20 in distilled water. The vehicle control group received the same vehicle without HBE. Donepezil was used as the positive control at 0.75 mg/kg and was prepared in the same vehicle. DPZ was administered orally once daily during the same 28-day treatment period.

#### 2.8.4 Passive avoidance test (PAT)

Contextual fear-associated learning and memory were evaluated using the passive avoidance test, based on a previously described method with minor modification [47]. The apparatus consisted of an illuminated chamber and a dark chamber of equal size (25 × 20 × 30 cm), separated by a guillotine door. During training, each mouse was first placed in the illuminated chamber with the door closed. After 10 s, the door was opened, and the mouse was allowed to enter the dark chamber. Once the mouse entered the dark compartment, the door was closed and a 2 s foot shock with a peak voltage of 500 V was delivered through the grid floor. Training was performed three times per day for five consecutive days from day 16 to day 20.

On day 21, scopolamine was injected 30 min before the retention test. During the test session, each mouse was placed in the illuminated chamber, and the time required to enter the dark chamber was recorded without delivery of foot shock. A longer step-through latency was considered to reflect better retention of the aversive experience. The maximum cut-off time was 180 s. The apparatus was cleaned with 70% ethanol between trials to reduce olfactory interference.

#### 2.8.5 Water Maze Test (WMT)

Spatial learning and memory were assessed using the Morris water maze test with minor modification of a previously reported protocol [48]. The test was performed in a circular white pool measuring 150 cm in diameter and 65 cm in height. The pool was filled with water to a depth of 50 cm, and the water temperature was maintained at 23–27 °C. A white circular escape platform measuring 10 cm in diameter and 45 cm in height was positioned 5 cm below the water surface.

Mice were trained twice daily for three consecutive days from day 25 to day 27. When a mouse located the hidden platform, it was allowed to remain on the platform for 10 s. If a mouse failed to find the platform within 180 s, it was gently guided to the platform and kept there for 10 s. On day 28, two test trials were conducted for each mouse with a 20 min interval between trials. Escape latency, defined as the time required to locate the submerged platform, was recorded, and the mean value of the two trials was used for analysis. Scopolamine was administered intraperitoneally 30 min before the test.

### 2.9 Characterization of bioactive compounds by UPLC-QTOF-MS/MS

For chemical profiling, the ethanol extract was dissolved in 70% methanol to a final concentration of 100 μg/mL and filtered through a 0.2 μm PTFE syringe filter before analysis. Chromatographic separation was performed on an Acquity UPLC I-Class system (Waters, Milford, MA, USA) using a BEH C18 column (150 × 2.1 mm, 1.7 μm; Waters). The autosampler temperature was maintained at 10 °C, and the column temperature was set to 40 °C. The mobile phase consisted of 0.1% formic acid in water as solvent A and 0.1% formic acid in acetonitrile as solvent B. The flow rate was maintained at 0.3 mL/min, and the injection volume was 2 μL. The gradient elution program was as follows: 0–5 min, 10–20% B; 5–25 min, 20–22% B; 25–45 min, 22–25% B; 45–65 min, 25–30% B; and 65–85 min, 30% B. Mass spectrometric analysis was carried out using a Xevo G2 QTOF mass spectrometer (Waters MS Technologies, Manchester, UK) equipped with an electrospray ionization source operated in negative-ion mode. Data-independent MS/MS acquisition was performed in MSE mode using a collision-energy ramp of 15–45 eV. The source conditions were as follows: capillary voltage, 2.5 kV; cone voltage, 45 V; source temperature, 120 °C; desolvation temperature, 350 °C; cone gas flow, 50 L/h; and desolvation gas flow, 800 L/h. Spectra were acquired in Continuum mode over an *m*/*z* range of 100–1500, with a scan time of 0.25 s and an interscan delay of 0.014 s. The resolving power was maintained above 20,000 FWHM. Sodium formate was used for mass calibration. Leucine enkephalin at *m*/*z* 554.2615 in negative-ion mode was infused at 200 pg/μL and 5 μL/min and used as the lock-mass reference every 10 s. Data acquisition and processing were performed using MassLynx version 4.1, and tentative compound assignment was carried out using UNIFI version 1.8.

### 2.10 Tentative identification using UNIFI and an in-house library

The chemical constituents of HBE were tentatively assigned using the UNIFI data-processing platform. The Traditional Chinese Medicine library included in UNIFI, containing more than 7500 natural-product entries, was expanded with hemp-related compounds collected from the literature up to 2020. Additional compound information was obtained from the Dictionary of Natural Products, SciFinder Scholar, and the Natural Product Activity and Species Source Database. Chemical structures were prepared using ChemBioDraw 15.0 and exported as individual.mol files before being incorporated into a custom in-house library. Chromatographic features were processed within a 1–85 min retention-time window. The intensity thresholds were set at 3000 counts for low-energy spectra and 300 counts for high-energy spectra, and the mass-error tolerance was set to ±5 ppm. Tentative assignments were made by comparing accurate mass values, predicted molecular formulas, and MS/MS fragmentation patterns with entries in the UNIFI Traditional Chinese Medicine library and the custom hemp-specific database.

### 2.11 *In silico* analysis

#### 2.11.1 Preparation of macromolecules and ligands

The crystal structures of acetylcholinesterase, butyrylcholinesterase, and monoamine oxidase-B were obtained from the RCSB Protein Data Bank using PDB IDs 4EY7, 5DYY, and 2V5Z, respectively [49], [50], [51]. Protein structures were prepared by removing crystallographic water molecules and heteroatoms, adding polar hydrogen atoms, and assigning partial charges to standard amino acid residues. The prepared structures were saved in PDBQT format for docking. Seven cannabinoids detected in HBE and evaluated experimentally in enzyme assays were selected for computational analysis. Target-specific reference ligands were also included: donepezil for AChE, 5HH for BuChE, and safinamide for MAO-B. Three-dimensional structures of the ligands were obtained from PubChem. Ligand structures were protonated, assigned partial charges, and converted to PDBQT format using OpenBabel [52]. Rotatable bonds were assigned to allow ligand flexibility during docking. Because CBDA and CBCA contain carboxylic acid groups, both neutral and monoanionic forms were evaluated. The anionic forms were generated by deprotonating only the carboxylic acid group while keeping the phenolic hydroxyl groups neutral, allowing the influence of protonation state on docking and GNINA rescoring to be examined.

#### 2.11.2 Molecular docking and consensus rescoring

Molecular docking was executed using AutoDock Vina version 1.2.0.52 with standard Vina parameters [53]. Docking grids were defined to cover the active-site regions of each target enzyme based on their corresponding co-crystallized ligands. The grid centers were set as follows: AChE, x = −14.43, y = −42.99, z = 28.15; BuChE, x = 3.93, y = −9.44, z = −13.05; and MAO-B, x = 52.37, y = 156.19, z = 28.77. Docking was performed with an exhaustiveness value of 16, a maximum of 20 binding modes, and an energy range of 4 kcal/mol. For each protein–ligand pair, ten candidate poses were generated, and the representative pose was selected by considering both the most favorable binding affinity and the lowest RMSD. To assess reproducibility, five independent docking runs were performed using different random seeds: 11111, 22222, 33333, 44444, and 55555. The pose with the most favorable binding energy was used as the reference, and RMSD values of the top poses from the remaining runs were calculated relative to this reference. To validate the docking protocol, native-ligand redocking was performed using the co-crystallized ligands E20 for AChE, 5HH for BuChE, and SAG for MAO-B. Each native ligand was redocked into its corresponding binding pocket. The resulting poses were inspected using Schrödinger Maestro [54] and BIOVIA Discovery Studio Visualizer (v4.5) [55] to evaluate ligand orientation and non-covalent interactions. Protein–ligand interaction fingerprints and 2D interaction diagrams were generated using ProLIF [56].

#### 2.11.3 GNINA CNN rescoring

The top-ranked Vina poses from each independent docking replicate were rescored using GNINA with convolutional neural network scoring enabled [57]. For each protein–ligand complex, the five Vina-derived poses were analyzed to obtain CNNaffinity and CNNscore values. Vina binding affinity, estimated inhibition constant, replicate-to-reference RMSD, CNNaffinity, and CNNscore were summarized as the mean ± standard deviation across the five docking replicates. Pose reproducibility was evaluated using an RMSD clustering threshold of 2.0 Å, and the dominant binding orientation was reported as the consensus cluster frequency out of five runs. For prioritized HBE-derived cannabinoids, representative complexes were selected from RMSD-converged clusters using agreement between Vina and GNINA scores. These complexes were used for subsequent molecular dynamics simulation and binding free-energy analysis. Data extraction and analysis were performed using custom in-house Python scripts. All successfully generated docking and rescoring results were retained for analysis without selective omission.

#### 2.11.4 Molecular dynamics simulations

The dynamic stability of selected protein–ligand complexes was evaluated using 100 ns molecular dynamics simulations performed with GROMACS v2025.3 [58]. The selected docked complexes were converted from PDB to GROMACS coordinate format (.gro) and used as starting structures. Each complex was placed in a periodic simulation box and solvated explicitly using the TIP3P water model, with at least 1.2 nm between the solute and the box boundary. Sodium and chloride ions were added to neutralize the system and to obtain a final salt concentration of 0.15 M. Energy minimization was first performed using the steepest descent algorithm to remove steric clashes and unfavorable contacts. The minimized systems were then equilibrated in two steps. First, NVT equilibration was conducted for 1 ns while gradually heating the system from 0 to 300 K. This was followed by 1 ns NPT equilibration at 1 bar to stabilize pressure and solvent density. After equilibration, unrestrained production MD simulations were conducted for 100 ns. Coordinates were saved every 10 ps, yielding 10,000 trajectory frames for each complex. Trajectories were analyzed using standard GROMACS tools to calculate RMSD, RMSF, radius of gyration, intermolecular hydrogen bonds, and protein–ligand contacts.

#### 2.11.5 MM-GBSA and MM-PBSA binding free-energy calculations

MM-GBSA and MM-PBSA calculations were performed to estimate the binding free energies of the selected protein–ligand complexes [59], [60]. The processed 100 ns production trajectories were used for energy calculations. Binding free energies were averaged over the full trajectory to obtain ensemble-based estimates of complex stability.

### 2.12 Statistical analysis

All *in vitro* data are expressed as the mean ± standard deviation from three independent experiments. Animal data are presented as the mean ± standard deviation for individual mice in each treatment group. Statistical analyses were performed using GraphPad Prism 8 for Windows (GraphPad Software, La Jolla, CA, USA). Differences among multiple groups were analyzed by one-way analysis of variance followed by Dunnett's multiple-comparisons test against the appropriate control group. All statistical tests were two-sided, and p < 0.05 was considered statistically significant.

## 3 Results

### 3.1 Comparative inhibitory activities of hemp part extracts on AChE, BuChE, MAO-B, and ABTS radical scavenging

To systematically evaluate the multi-target pharmacological potential of ethanol extracts prepared from five anatomical parts of hemp (root, stem, bark, leaf, and seed) were subjected to comparative analysis for their inhibitory activities against AChE, BuChE, and MAO-B, as well as their ABTS radical scavenging capacities. The inhibitory effects of each extract on AChE and BuChE were evaluated over a concentration range of 0.01–1 mg/ml. All extracts inhibited AChE activity in a dose-dependent manner (Fig. 1a). Among them, the stem, bark, leaf, and seed extracts produced more than 60% inhibition of AChE activity, whereas the root extract showed less than 50% inhibition within the tested concentration range (Fig. 1a). The root, stem, bark, leaf, and seed extracts exhibited IC_50_ values of >1.0, 0.53, 0.56, 0.54, and 0.28 mg/mL, respectively, for AChE inhibition (Fig. 1e). Similarly, all extracts inhibited BuChE activity in a dose-dependent manner (Fig. 1b). Among the five extracts, the bark and leaf extracts showed the strongest BuChE inhibitory effects, each producing more than 80% inhibition at 0.1 mg/mL. The root, stem, bark, leaf, and seed extracts exhibited IC_50_ values of 0.88, 0.06, 0.01, 0.01, and 0.13 mg/mL, respectively, for BuChE inhibition (Fig. 1e). The inhibitory effects of the five hemp extracts on MAO-B were also evaluated over a concentration range of 0.01 – 0.2 mg/ml.

**Fig. 1:**
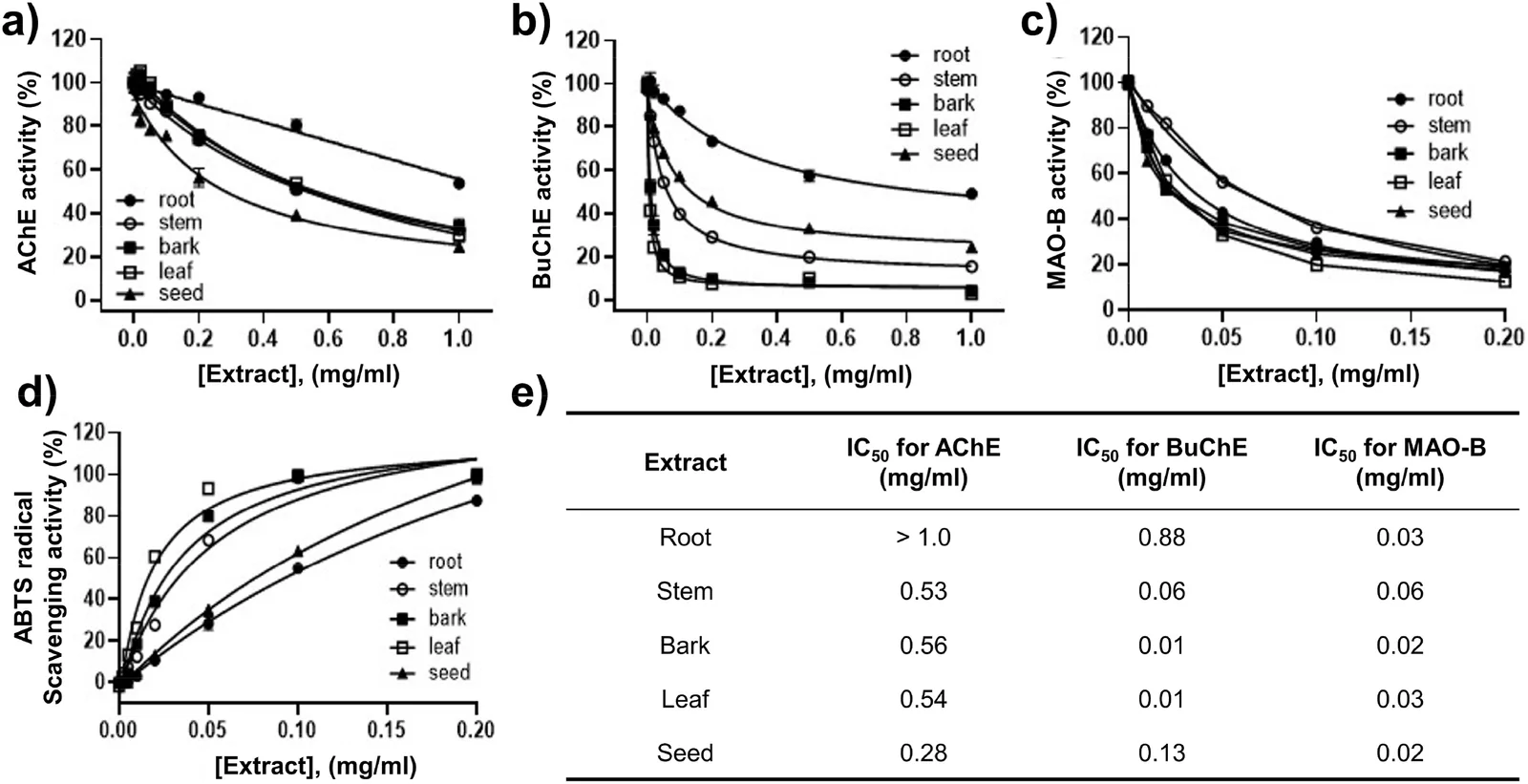
a) AChE inhibitory activity of each extract, b) BuChE inhibitory activity of each extract, c) MAO-B inhibitory activity of each extract, d) ABTS radical scavenging activity of each extract, e) IC_50_ values of the extracts against AChE, BuChE, and MAO-B. Data are presented as the mean ± standard deviation from three independent experiments.

All extracts inhibited MAO-B activity in a concentration-dependent manner, with IC_50_ values ranging from 0.02 to 0.06 mg/ml (Fig. 1c and e). These results indicate that the stem, bark, leaf, and seed extracts showed relatively comparable AChE inhibitory activity, whereas the bark and leaf extracts exhibited markedly stronger BuChE inhibition than the other extracts. Further, for significant MAO-B inhibition was demonstrated by bark and leaf extracts. Based on these results we believe that bark and leaf extracts contain relatively higher levels of bioactive constituents associated with cholinesterase and MAO-B inhibition, supporting their potential relevance for further investigation in cognitive dysfunction-related models. Because reference inhibitors were not tested in parallel in the enzyme inhibition assays, the IC_50_ values should be interpreted as comparative potencies within various hemp extracts rather than as direct potency benchmarks against standard cholinesterase or MAO-B inhibitors.

The antioxidant activity of the five hemp extracts was assessed by measuring their ABTS radical scavenging capacity within a concentration range of 0.02 – 0.2 mg/ml (Fig. 1d). A concentration-dependent increase in ABTS radical scavenging activity was evident in all extracts. Notably, the stem, bark, and leaf extracts achieved complete scavenging of ABTS radical at 0.1 mg/ml. Among the tested extracts, the leaf extract exhibited the highest ABTS radical scavenging potential, with subsequent activity observed in bark, stem, seed, and root extracts.

### 3.2 Effects of hemp part extracts on H_2_O_2_ induced intracellular ROS and SOD activity in SH-SY5Y neuronal cells

The 30 min treatment of SH-SY5Y cells with 400 μM H_2_O_2_ significantly increased intracellular ROS levels by approximately 2.2-fold as compared to H_2_O_2_–untreated control (Fig. 2a). Among the five extracts, the leaf, bark, root, and seed extracts significantly reduced H_2_O_2_–induced ROS accumulation, whereas the stem extract did not show a notable effect (Fig. 2a). For the determination of SOD activity, SH-SY5Y cells were treated with 0.2 μg/ml of each hemp extract in the presence of 200 μM H_2_O_2_ for 24 h. The SOD activity in untreated cells was considered 100% and relative SOD activities were determined for the hemp extract treated cells. The bark and leaf treated extracts demonstrated significant recovery in SOD activity compared to other extracts.

**Fig. 2:**
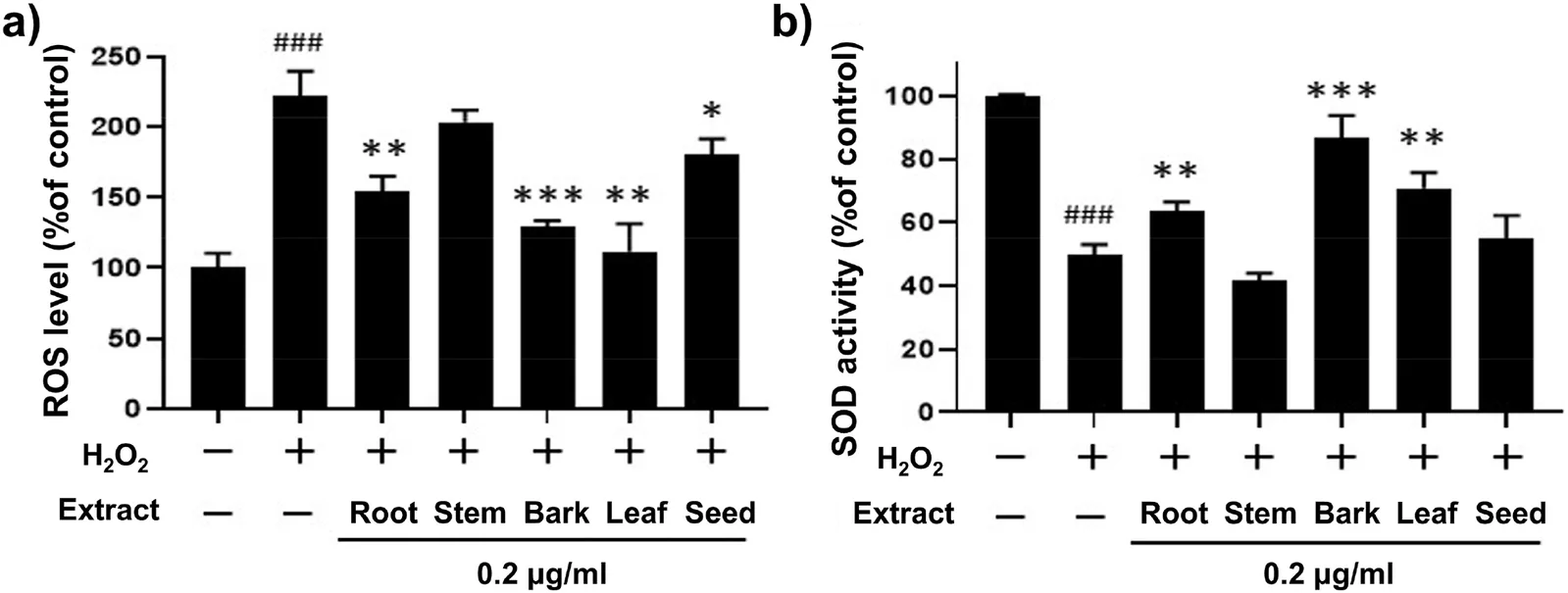
a) Intracellular ROS levels were measured using the H_2_DCFDA (25 μM) fluorescent probe after treatment of SH-SY5Y cells with each extract (0.2 μg/ml) and H_2_O_2_ (400 μM), b) SOD activity was determined using the xanthine oxidase/WST-1 assay by treating SH-SY5Y cells with each extract (0.2 μg/ml) and H_2_O_2_ (200 μM). Data are presented as the mean ± standard deviation from three independent experiments. ###p < 0.001 versus the normal control group; *p < 0.05, **p < 0.01, and ***p < 0.001 versus the hydrogen peroxide-treated group.

**Fig. 3:**
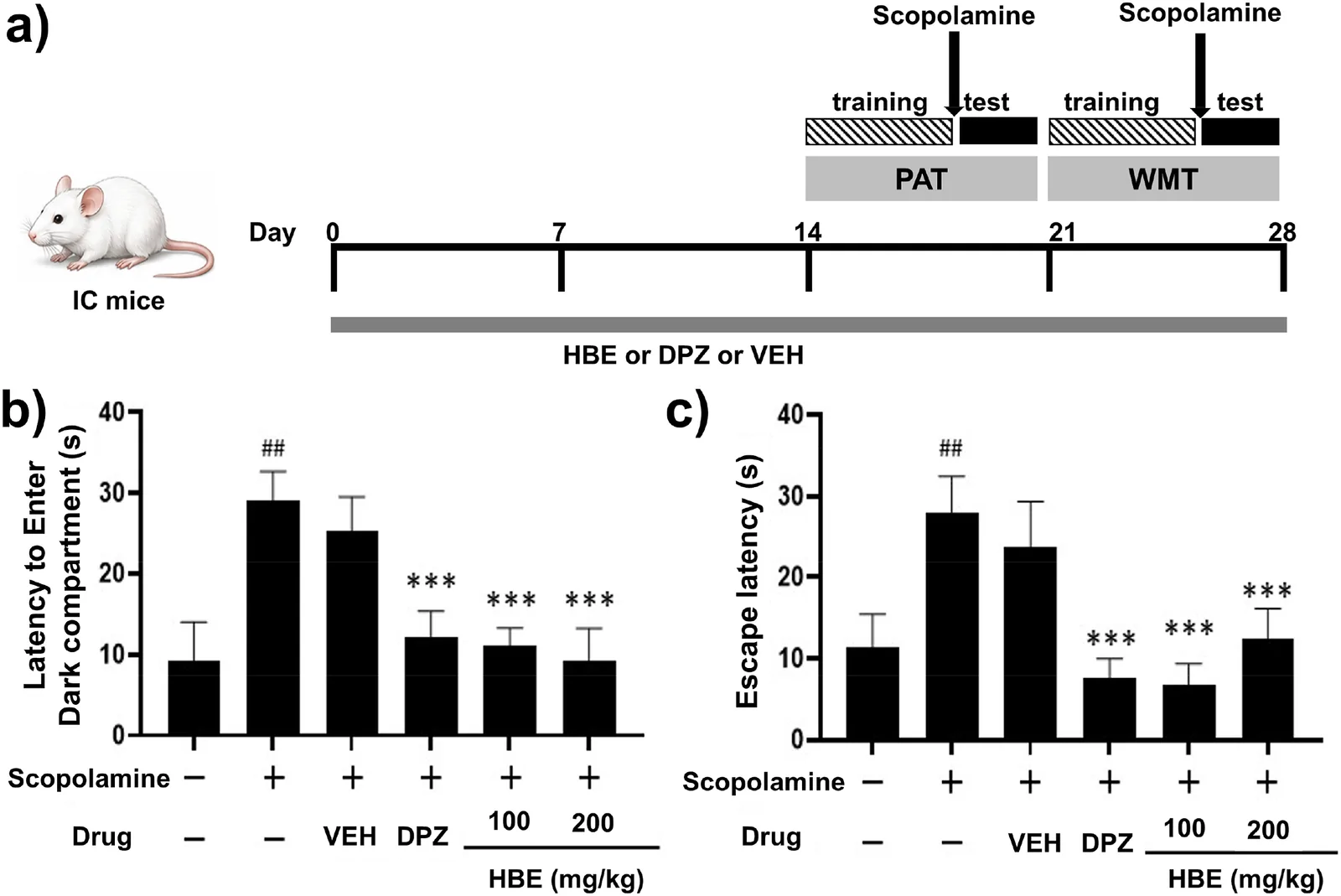
ICR mice were randomly divided into six groups (n = 5), including No treatment, Scopolamine, Scopolamine-vehicle (VEH), Scopolamine-donepezil (DPZ; 0.75 mg/kg), and Scopolamine-HBE (100 or 200 mg/kg). a) Experimental protocol for HBE, vehicle, or donepezil administration and scopolamine-induced cognitive impairment. The HBE, DPZ, or VEH was administered orally for days 0 – 28. Scopolamine (1 mg/kg) was injected intraperitoneally on day 21 for PAT and day 28 for WMT to induce short-term cognitive impairment in ICR mice, b) Outcomes of the passive avoidance test (PAT). Step-through latency was defined as the duration each mouse took to move from the illuminated aversive chamber to the electrified platform (n = 5 per group), c) Outcomes from the water maze test (WMT). The escape latency, or time required for each mouse to locate the hidden platform, was measured, and outcomes are presented as mean ± standard deviation. (##p < 0.01 vs normal group; ***p < 0.001 vs scopolamine-VEH group).

Based on the comparative *in vitro* screening, hemp bark extract (HBE) was selected for further *in vivo* evaluation because it showed a balanced multi-target activity profile, including strong AChE and BuChE inhibition, significant MAO-B inhibition, and effective antioxidant activity. Although both bark and leaf extracts showed strong enzyme inhibitory effects, HBE also markedly reduced H_2_O_2_ induced intracellular ROS accumulation and significantly restored SOD activity in SH-SY5Y neuronal cells. Overall, HBE demonstrated robust neuroprotective effects against oxidative stress-induced injury in neuronal cells. Therefore, HBE was considered the most suitable candidate for subsequent *in vivo* studies aimed at evaluating its protective effects against cognitive dysfunction.

### 3.3 Preventive effects of HBE on scopolamine-induced short-term cognitive dysfunction in mice

To investigate the potential cognitive benefits of HBE *in vivo*, we evaluated the preventive effects of HBE in a scopolamine-induced mouse model of short-term cognitive dysfunction. Male ICR mice were orally administered with vehicle, donepezil (DPZ; 0.75 mg/kg), or HBE (100 or 200 mg/kg) once daily for 28 consecutive days. Scopolamine (1 mg/kg, i.p.) was administered before behavioral testing to induce acute cholinergic impairment, and cognitive performance was assessed using the passive avoidance and water maze tests (Fig. 3a). In the PAT conducted on day 21, mice in the scopolamine-vehicle group exhibited a significantly prolonged latency (approximately 3.2-fold increase) to enter the dark compartment compared to the control group, confirming successful induction of memory impairment (Fig. 3b). Oral pre-treatment with HBE at both 100 and 200 mg/kg significantly reduced the scopolamine-induced step-through latency, indicating effective prevention of memory impairment. The reference drug donepezil (0.75 mg/kg) produced an equivalent reduction in latency (p < 0.001).

In the Morris water maze test (WMT) conducted on day 28, scopolamine-treated animals demonstrated a 2.5-fold prolongation of escape latency relative to untreated controls, confirming robust spatial memory impairment (Fig. 3c). Pre-administration of HBE at both 100 and 200 mg/kg significantly shortened escape latency, indicating prevention of hippocampus-dependent spatial memory deficits. Donepezil likewise produced significant improvements. Notably, both doses of HBE produced comparable and robust efficacy, with no statistically significant dose-response difference between the 100 and 200 mg/kg groups, suggesting that 100 mg/kg may represent a near-maximal effective dose in this model. Collectively, these results provide compelling *in vivo* evidence that HBE prevents scopolamine-induced short-term cognitive dysfunction, exerting protective effects on both contextual fear-associated memory and spatial learning and memory.

### 3.4 Identification of bioactive compounds in HBE by UPLC-QTOF-MS/MS and their inhibitory activities against AChE, BuChE, and MAO-B

To characterize the chemical constituents responsible for the observed pharmacological activities of HBE, the extract was subjected to UPLC-QTOF-MS/MS analysis in negative-ion electrospray ionization mode. A total of 26 compounds were tentatively identified within a retention time window of 0 – 8 min (Fig. 4a), based on accurate mass measurements (mass error ≤ ± 5 ppm), molecular formula assignments, and MS/MS fragmentation pattern matching against the UNIFI Traditional Chinese Medicine database supplemented with a custom in-house hemp-specific compound library (Table S1, Fig. S1). Three compounds (peaks 4, 12, and 13) could not be matched to known library entries and were provisionally annotated by empirical molecular formula only.

**Fig. 4:**
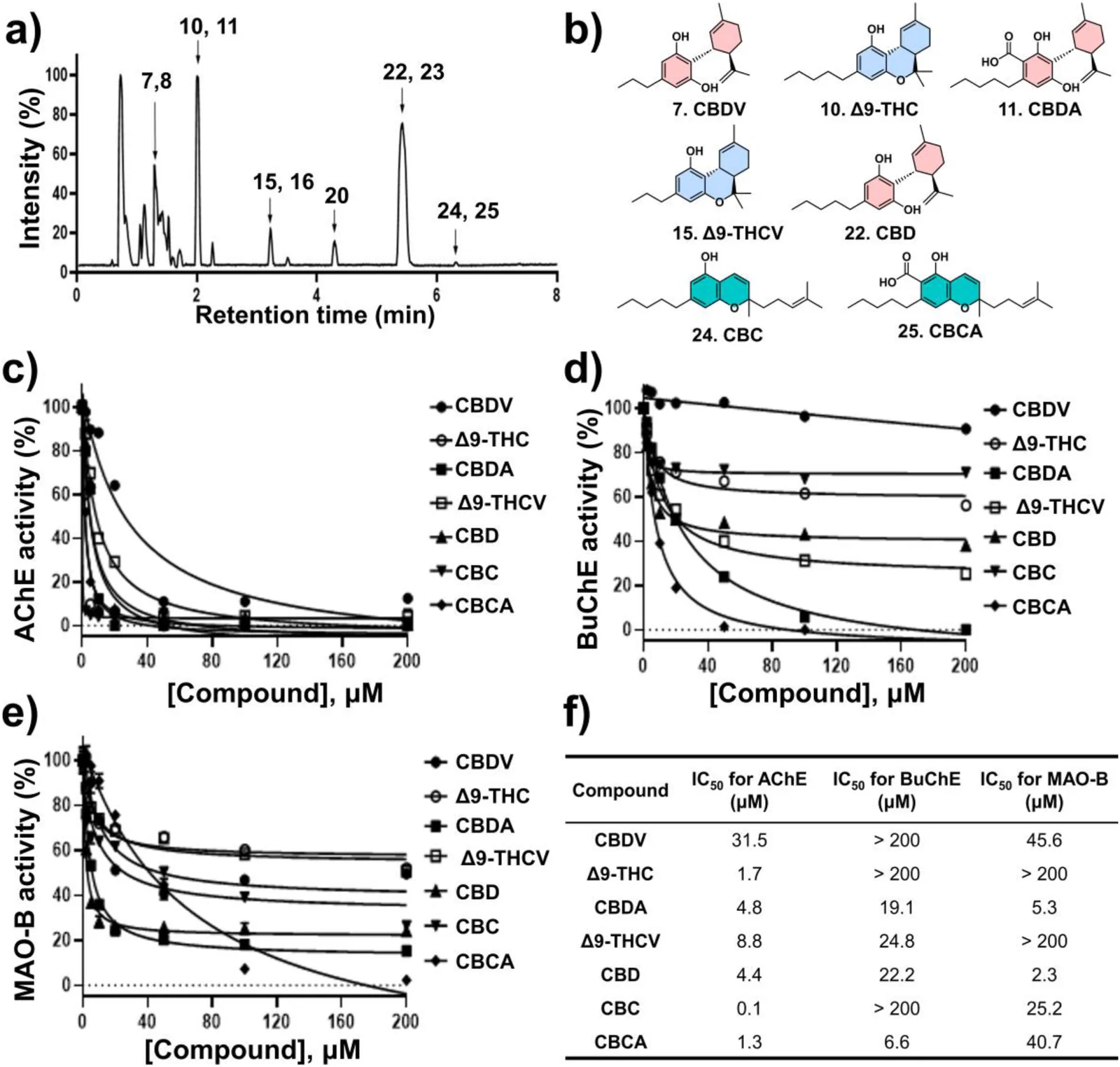
a) UPLC-QTOF-MS total ion chromatogram of HBE in negative ionization mode, b) Principal cannabinoid derivatives of HBE identified by analyzing the MS/MS fragmentation pattern matching against the UNIFI Traditional Chinese Medicine database, c) Effects of cannabinoids on AChE, d) Effects of cannabinoids on BuChE, e) Effects of cannabinoids on MAO-B, f) The IC_50_ of cannabinoids for AChE, BuChE, and MAO-B. Results are presented as mean ± SD from three independent experimental replicates.

Among the identified constituents, based on characteristic fragmentation patterns seven cannabinoids (Fig. 4b) were unambiguously recognized, including cannabidivarin (CBDV, peak 7; *m*/*z* [M − H]^-^ 285.1854), Δ9-tetrahydrocannabinol (Δ9-THC, peak 10; *m*/*z* 313.2177), CBDA (peak 11; *m*/*z* 357.2074), Δ9-tetrahydrocannabivarin (Δ9-THCV, peak 15; *m*/*z* 285.1868), CBD (peak 22; *m*/*z* 313.2167), cannabichromene (CBC, peak 24; *m*/*z* 313.2178), and CBCA (peak 25; *m*/*z* 357.2075). In addition, phenolic compounds (β-gentiobiose, syringic acid-O-hexoside), acidic cannabinoids (cannabinolic acid, CBNA; cannabidivarinic acid, CBDVA; tetrahydrocannabivarinic acid, THCVA; Δ9-tetrahydrocannabinolic acid A, THCAA), and polyunsaturated fatty acids (gamma-linolenic acid, alpha-linolenic acid, linoleic acid, hexadecanoic acid) were also identified.

We procured the seven cannabinoids identified in the HBE extract (Fig. 4b) to understand their individual contributions to the multi-target inhibitory profile of HBE. The seven representative cannabinoids (CBDV, Δ9-THC, CBDA, Δ9-THCV, CBD, CBC, and CBCA) were evaluated for their inhibitory activities against AChE, BuChE, and MAO-B using *in vitro* enzymatic assays. For AChE inhibition, all seven cannabinoids demonstrated concentration-dependent inhibitory activity within 0 – 200 μM, with IC_50_ values spanning 0.1 to 31.5 μM (Fig. 4c and f). CBC exhibited the most potent AChE inhibitory activity with IC_50_ value of 0.1 μM followed by CBCA (IC_50_ = 1.3 μM), Δ9-THC (IC_50_ = 1.7 μM), CBD (IC_50_ = 4.4 μM), CBDA (IC_50_ = 4.8 μM), Δ9-THCV (IC_50_ = 8.8 μM). The CBDV (IC_50_ = 31.5 μM) was found to show least potent AChE inhibitory activity.

For BuChE inhibition, three (CBDV, Δ9-THC, and CBC) of the seven cannabinoids exhibited poor activity indicated by IC_50_ values > 200 μM (Fig. 4d and f). CBDA, Δ9-THCV, and CBD demonstrated comparable activities with the IC_50_ values of 19.1, 24.8, and 22.2 μM, respectively. CBCA demonstrated the most potent BuChE inhibitory activity with the IC_50_ value of 6.6 μM. For MAO-B inhibition, five of the seven cannabinoids produced concentration-dependent inhibitory effects (Fig. 4e and f). CBD demonstrated the strongest MAO-B inhibitory activity with the IC_50_ value of 2.3 μM, followed by CBDA with the IC_50_ value of 5.3 μM. CBC, CBCA, and CBDV were also found to MAO-B activity but with relatively higher IC_50_ values of 25.2, 40.7, and 45.6 μM, respectively. Notably, Δ9-THC and Δ9-THCV, the structural isomers of CBD and CBDV, respectively did not show significant MAO-B inhibitory activity. Overall, the results indicate that several cannabinoids present in HBE possess multi-target inhibitory potential against enzymes associated with cognitive dysfunction. Among the tested compounds, CBD, CBDA, and CBCA showed the most relevant polypharmacological profiles by inhibiting AChE, BuChE, and MAO-B to various extents. CBCA exhibited strong dual cholinesterase inhibition, whereas CBD and CBDA showed more balanced activity against AChE and MAO-B. These findings suggest that the neuroprotective effects of HBE may arise from the combined multi-target actions of multiple cannabinoids rather than from a single active constituent.

### 3.5 Computational analysis of identified HBE constituents with various enzymes

Fig. 4e and f

The docking workflow was first evaluated by native-ligand redocking before analysis of the HBE-derived cannabinoids. The co-crystallized ligands E20, 5HH, and SAG were redocked into the active sites of AChE, BuChE, and MAO-B, respectively. The resulting poses showed excellent agreement with the crystallographic orientations, with RMSD values of 0.02 ± 0.01 Å for E20–AChE, 0.05 ± 0.03 Å for 5HH–BuChE, and 0.54 ± 0.71 Å for SAG–MAO-B (Table S3). Because all RMSD values were below 2.0 Å, the docking setup was considered reliable for evaluating the binding modes of the identified cannabinoids.

To rationalize the enzyme inhibitory activities of the cannabinoids identified in HBE, molecular docking and GNINA CNN-based rescoring were performed against AChE, BuChE, and MAO-B. The seven cannabinoids, CBDV, Δ9-THC, CBDA, Δ9-THCV, CBD, CBC, and CBCA, were docked into the active-site regions (Table S2) of the three enzymes, and the predicted binding energies and estimated *Ki* values (Table 1, Fig. 5), docking reproducibility (Table S3), CNNaffinity and CNNscore values (Table S4) were compared. The reproducibility of the docking protocol was supported by the RMSD and consensus analysis of five independent docking replicates. All enzyme–ligand pairs showed a consensus of 5/5 using a 2.0 Å cutoff (Table S3), indicating that the top-ranked docking poses were reproducibly generated across independent runs. The docking poses showed strong reproducibility across five independent runs, with RMSD values of 0.01 – 1.61 Å for AChE, 0.01 – 0.64 Å for BuChE, and 0.01 – 0.37 Å for MAO-B, and all enzyme–ligand pairs showed 5/5 consensus clustering using a 2.0 Å cutoff. Although a few systems, such as CBDA with AChE and CBC or CBCA with BuChE, showed higher RMSD variability, they still maintained full consensus clustering, suggesting that the predicted binding regions were consistent across replicates.

**Table 1:** Docking-derived binding energies (ΔG, kcal/mol) and estimated inhibition constants (*K*_*i*_, μM) for ligands docked against AChE, BuChE, and MAO-B in five independent runs.

Ligand	AChE		BuChE		MAOB	
	ΔG (kcal/mol)	*K*_*i*_ (μM)	ΔG (kcal/mol)	*K*_*i*_ (μM)	ΔG (kcal/mol)	*K*_*i*_ (μM)
**CBDV**	−8.85 ± 0.01	0.33 ± 0.01	−7.95 ± 0.01	1.48 ± 0.02	−8.94 ± 0.02	0.28 ± 0.01
**Δ9-THC**	−10.29 ± 0.04	0.03 ± 0.00	−8.63 ± 0.02	0.47 ± 0.02	−8.34 ± 0.01	0.77 ± 0.02
**CBDA**	−8.70 ± 0.11	0.42 ± 0.08	−8.67 ± 0.02	0.44 ± 0.01	−7.91 ± 0.16	1.64 ± 0.51
**Δ9-THCV**	−9.75 ± 0.00	0.07 ± 0.00	−8.67 ± 0.02	0.44 ± 0.02	−7.69 ± 0.04	2.31 ± 0.16
**CBD**	−9.21 ± 0.01	0.18 ± 0.00	−8.55 ± 0.02	0.54 ± 0.02	−7.42 ± 0.11	3.69 ± 0.76
**CBC**	−9.60 ± 0.01	0.09 ± 0.00	−8.42 ± 0.11	0.69 ± 0.13	−9.22 ± 0.02	0.17 ± 0.01
**CBCA**	−9.66 ± 0.02	0.08 ± 0.00	−8.46 ± 0.05	0.63 ± 0.05	−9.10 ± 0.09	0.21 ± 0.04

**Fig. 5:**
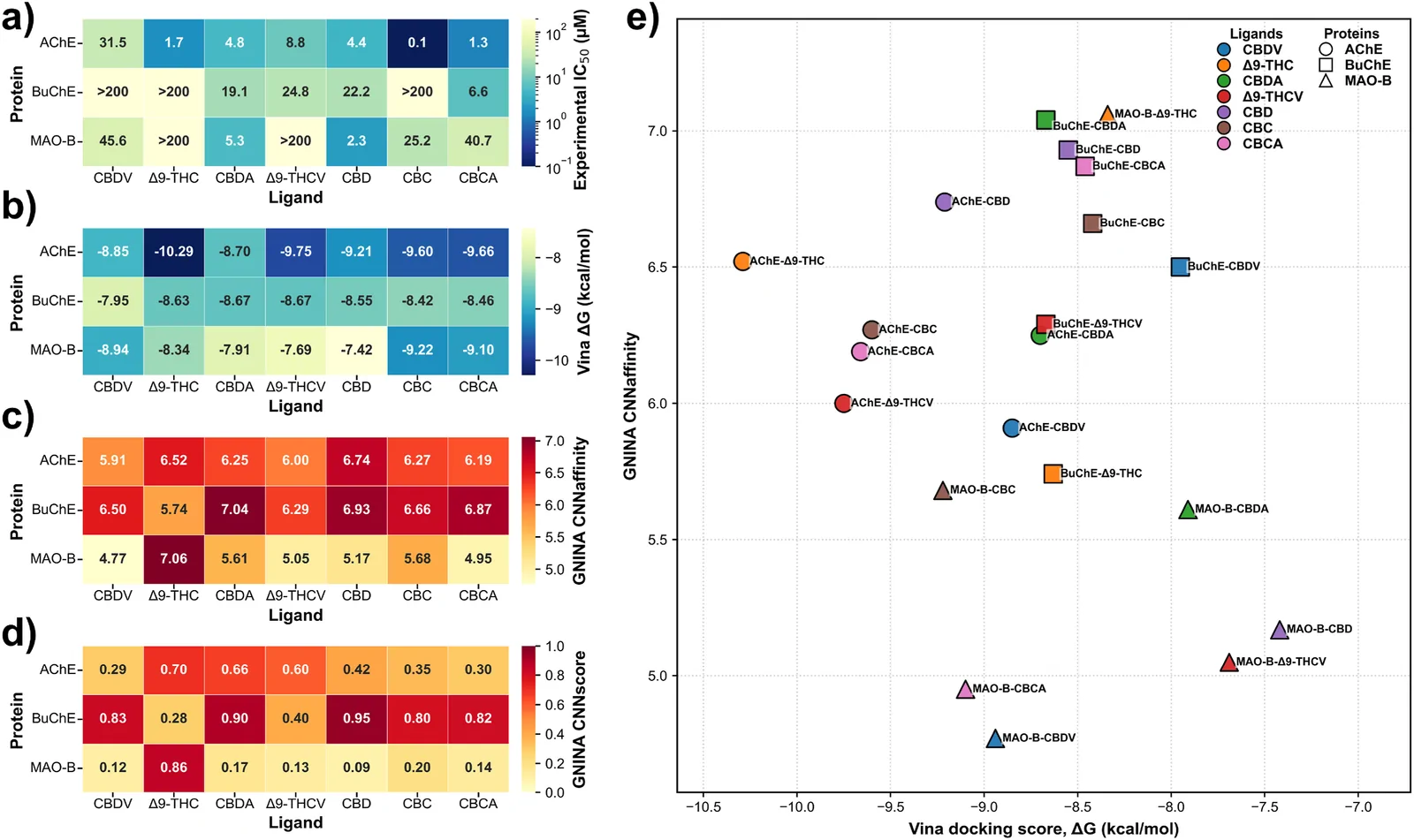
a) Experimental IC_50_ heatmap, b) AutoDock Vina binding energy heatmap, c) GNINA CNNaffinity heatmap, d) GNINA CNNscore heatmap, and e) scatter plot comparing Vina docking scores with GNINA CNNaffinity values.

To rationalize the enzyme inhibitory activities of the cannabinoids identified in HBE, molecular docking and GNINA CNN-based rescoring were performed against AChE, BuChE, and MAO-B. The seven cannabinoids, CBDV, Δ9-THC, CBDA, Δ9-THCV, CBD, CBC, and CBCA, were docked into the active-site regions (Table S2) of the three enzymes, and the predicted binding energies and estimated *Ki* values (Table 1, Fig. 5), docking reproducibility (Table S3), CNNaffinity and CNNscore values (Table S4) were compared. The reproducibility of the docking protocol was supported by the RMSD and consensus analysis of five independent docking replicates. All enzyme–ligand pairs showed a consensus of 5/5 using a 2.0 Å cutoff (Table S3), indicating that the top-ranked docking poses were reproducibly generated across independent runs. Most RMSD values were low, further supporting stable pose convergence. Although a few systems, such as CBDA with AChE and CBC or CBCA with BuChE, showed higher RMSD variability, they still maintained full consensus clustering, suggesting that the predicted binding regions were consistent across replicates.

AutoDock Vina docking showed that all seven cannabinoids formed energetically favorable complexes with AChE, BuChE, and MAO-B. For AChE, Δ9-THC showed the most favorable docking score (ΔG = −10.29 ± 0.04 kcal/mol, Table 1), followed by Δ9-THCV, CBCA, CBC, and CBD. This trend partially agreed with the experimental AChE inhibition data (Fig. 5a), in which CBC, CBCA, and Δ9-THC were among the most active compounds. In particular, CBC showed the strongest AChE inhibition experimentally, whereas CBCA and Δ9-THC also exhibited strong activity. These results suggest that the favorable AChE docking scores and GNINA analysis of CBC, CBCA, Δ9-THC, and CBD are consistent with their experimentally observed AChE inhibitory effects (Fig. 5b–5d).

For BuChE, the docking scores were relatively similar (ΔG = −8.67 to −7.95 kcal/mol, Fig. 5b) across the cannabinoid series, indicating that Vina alone did not clearly separate experimentally active and weakly active compounds. However, the GNINA results provided additional support for several BuChE-active cannabinoids (Table S4). CBDA, CBD, and CBCA showed high CNNaffinity values (*K*_*d*_ = 7.04, 6.93, 6.87) and high CNNscore (0.90, 0.95, and 0.82) values, indicating favorable predicted binding and reliable pose confidence (Fig. 5c and d). These compounds also showed measurable BuChE inhibitory activity *in vitro*, with CBCA being the most potent BuChE inhibitor among the tested cannabinoids. Therefore, the combined Vina and GNINA analyses support the relevance of CBCA, CBDA, and CBD as BuChE-interacting constituents of HBE.

For MAO-B, the computational results showed partial agreement with the experimental inhibition profile. Experimentally, CBD and CBDA exhibited the strongest MAO-B inhibitory activities, whereas CBC and CBCA also showed measurable, although weaker, inhibition. Consistent with the ability of these compounds to interact with MAO-B, Vina docking (Fig. 5b) predicted favorable binding energies for CBC (ΔG = −9.22 ± 0.02 kcal/mol) and CBCA (ΔG = −9.10 ± 0.09 kcal/mol), and GNINA rescoring (Fig. 5c and d) also supported binding-site compatibility for CBDA, CBD, CBC, and CBCA. Although the exact computational ranking did not fully reproduce the experimental IC_50_ order, the combined docking and GNINA results indicate that these experimentally active cannabinoids can occupy the MAO-B binding pocket with favorable predicted interactions.

To evaluate whether the docked poses remained stable under dynamic conditions, 100 ns molecular dynamics simulations were performed for selected cannabinoid complexes with AChE, BuChE, and MAO-B (Fig. 6, Fig. S2 and S3). Structural stability was assessed using protein root-mean-square deviation (RMSD), root-mean-square fluctuation (RMSF), number of protein–ligand contacts, radius of gyration, hydrogen-bond occupancy, and minimum protein–ligand distance profiles. Overall, the simulated complexes maintained stable protein conformations and compact structures throughout the production runs, as reflected by low RMSD fluctuations and narrow Rg distributions.

**Fig. 6:**
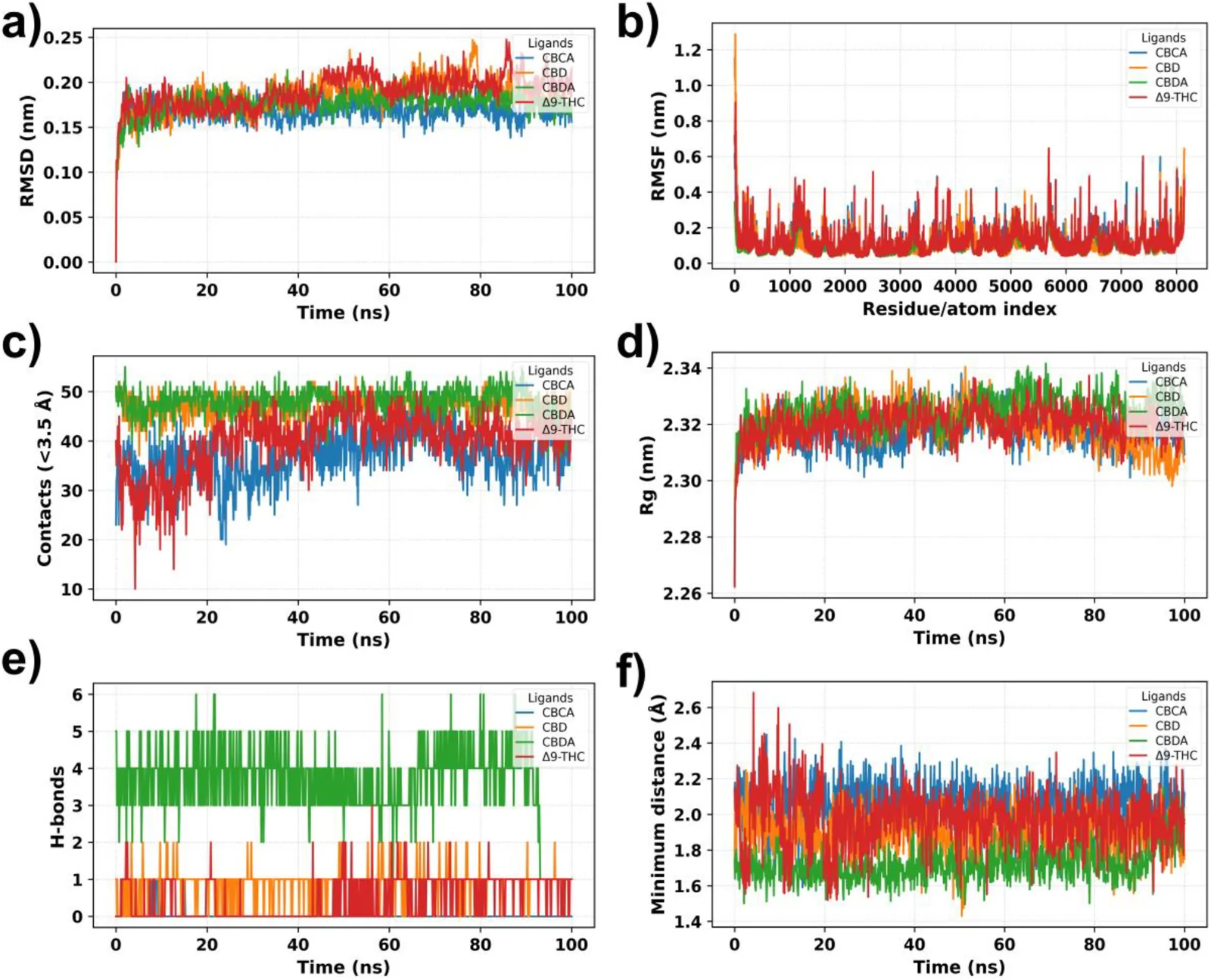
MD trajectory analysis of AChE complexes with CBCA, CBD, CBDA, and Δ9-THC over 100 ns. The plots show: a) root-mean-square deviation (RMSD), b) root-mean-square fluctuation (RMSF), c) protein–ligand contacts within 3.5 Å, d) radius of gyration (Rg), e) hydrogen-bond count, and f) average minimum protein–ligand distance.

For the AChE (Fig. 6, Table S5) complexes, all four ligands (CBCA, CBD, CBDA, and Δ9-THC) produced stable trajectories, with average protein RMSD values ranging from 0.170 ± 0.01 nm for CBCA to 0.201 ± 0.01 nm for Δ9-THC. The Rg values remained highly consistent at approximately 2.32 nm. Among the AChE complexes, CBDA showed the most favorable dynamic interaction profile, with the lowest RMSF value (0.082 ± 0.046 nm), the highest hydrogen-bond count (3.405 ± 1.227), and the shortest average minimum protein–ligand distance (1.748 ± 0.107 Å). CBD also maintained extensive contacts with AChE, showing 47.363 ± 1.972 contacts within 3.5 Å and an average minimum distance of 1.886 ± 0.109 Å. In contrast, CBCA showed fewer contacts and no persistent hydrogen-bond occupancy, although the RMSD and Rg values indicated that the complex remained structurally stable. These results suggest that CBDA and CBD form more persistent short-range interactions with AChE during MD simulation, whereas CBCA is mainly stabilized by nonpolar contacts.

The BuChE complexes also remained stable over 100 ns (Table S5, Fig. S2). CBD showed the lowest protein RMSD among the BuChE complexes (0.156 ± 0.007 nm) and the highest number of protein–ligand contacts (44.495 ± 3.345), suggesting stable accommodation within the BuChE binding site. CBCA and CBDA showed comparable RMSD values of 0.194 ± 0.013 and 0.193 ± 0.012 nm, respectively, while Δ9-THC showed a lower RMSD of 0.168 ± 0.010 nm. The average Rg values remained between 2.321 and 2.353 nm. Although persistent hydrogen bonding was limited in the BuChE systems, CBCA and Δ9-THC showed low but detectable hydrogen-bond occupancies, whereas CBD and CBDA were mainly stabilized by hydrophobic and van der Waals contacts.

For MAO-B, the MD profiles showed ligand-dependent differences in complex stability (Table S5, Fig. S3). CBD and CBDA produced the most stable protein RMSD values, with averages of 0.168 ± 0.019 and 0.148 ± 0.022 nm, respectively. In contrast, CBC, CBCA, and Δ9-THC showed higher RMSD values, particularly CBCA and Δ9-THC, which reached 0.362 ± 0.021 and 0.388 ± 0.011 nm, respectively. Despite these differences, Rg values remained within a narrow range of 2.356–2.387 nm. CBDA showed the strongest dynamic interaction profile with MAO-B, exhibiting the highest hydrogen-bond count (3.070 ± 0.430) and the shortest minimum distance (1.644 ± 0.087 Å). CBD also showed a favorable profile, with 50.232 ± 1.515 contacts and 2.234 ± 0.554 hydrogen bonds. These results support the stable binding of CBDA and CBD in the MAO-B active site, consistent with their experimentally observed MAO-B inhibitory activity.

Representative post-MD 3D poses and the corresponding 2D interaction maps were examined together to define the residue-level interaction patterns that stabilized the selected cannabinoid–enzyme complexes after 100 ns MD simulation (Fig. 7 and Fig. S4–S7). In AChE, CBCA, CBD, and CBDA remained positioned within the binding-site and were stabilized by a combination of hydrophobic, aromatic, van der Waals, and polar interactions. CBCA interacted with S284 (3.21 Å), E283 (3.39 Å), Y332 (3.42 Å), Y121 (3.50–3.56 Å), L280 (3.58 Å), W277 (3.62 Å, hydrophobic; 4.11 Å, π–π stacking), and F288 (3.70 Å) by either van der Waals or hydrophobic interactions (Fig. 7a and S5a). CBD (Fig. 7b and S5b) showed hydrogen-bonding interaction with Y328 (2.04 Å). The other interactions mostly included van der Waals and hydrophobic interactions with H438 (3.13 Å), S122 (3.17 Å), Y121 (3.25 Å), W83 (3.61 Å), W277 (3.70 Å), and F288 (3.80 Å). CBDA (Fig. 7c and S5c) displayed the most evident polar interaction pattern among the AChE ligands, forming a hydrogen-bonds with G118 (2.22 Å), S200 (2.41 Å) and Y328 (2.13 Å). The other interactions included van der Waals and hydrophobic interactions with W83 (3.11 Å), Y121 (3.50–3.77 Å), and F329 (3.71 Å). The Δ9-THC–AChE complex (Fig. S4a and S5d) was similarly dominated by aromatic and hydrophobic contacts, including Y328 (3.19 Å), F329 (3.44 Å), Y332 (3.68 Å), Y121 (3.74 Å), and W277 (4.20 Å, π–π stacking). These interaction patterns agree with the MD trajectory results, in which CBDA and CBD showed stronger short-range contact and hydrogen-bonding behavior than CBCA.

**Fig. 7:**
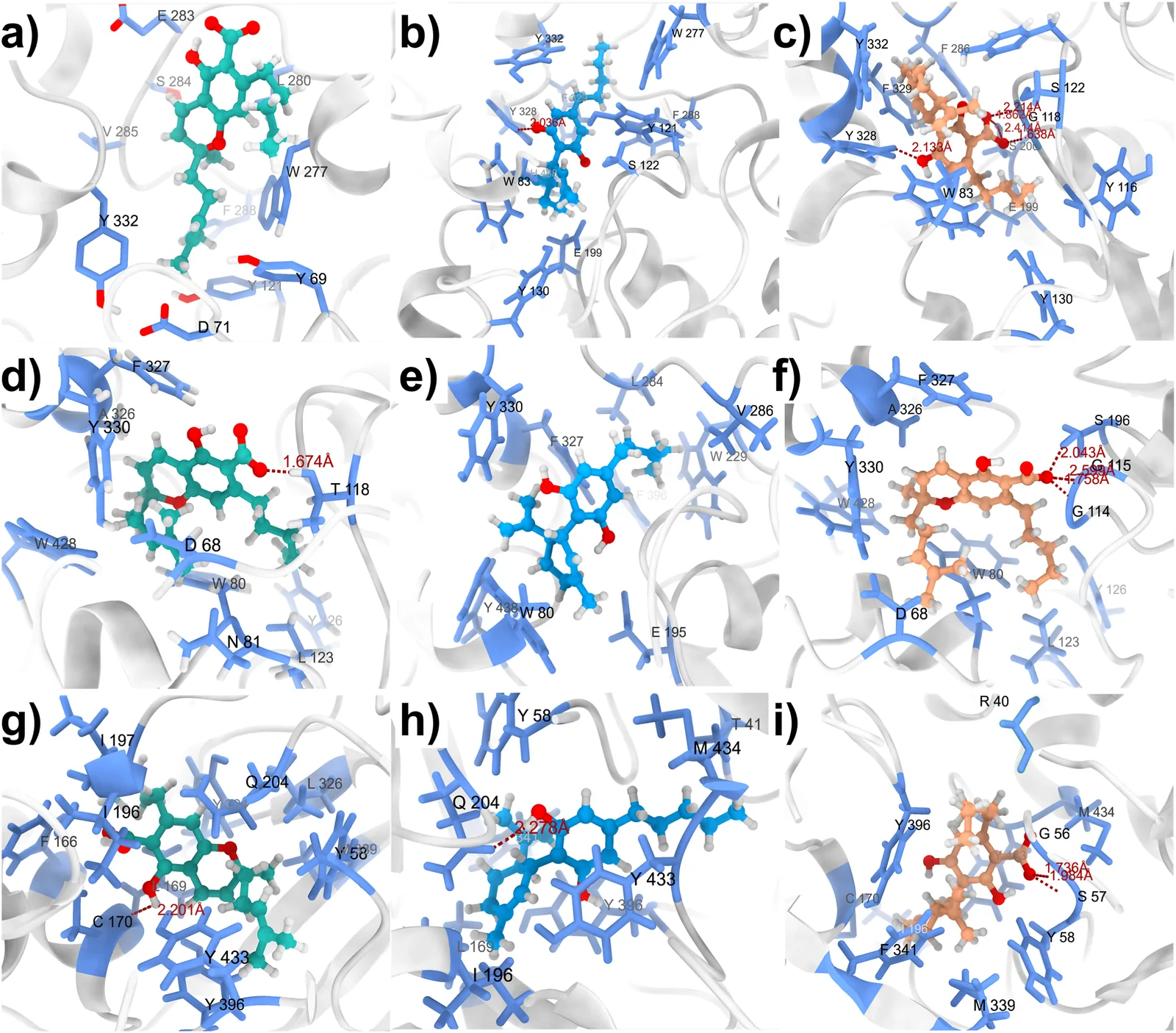
Ligand–protein interactions analyzed for ligands a) CBCA, b) CBD, c) CBDA with AChE, d) CBCA, e) CBD, f) CBDA with BuChE, g) CBCA, h) CBD, i) CBDA with MAO-B upon 100 ns MD simulation (interacting amino acids, blue; CBCA, teal; CBD, sky blue; CBDA, peach; red dotted lines, hydrogen-bonding interactions).

In CBCA–BuChE complex (Fig. 7d and S6a), CBCA formed a clear hydrogen-bond interaction with T118 (1.67 Å), along with hydrophobic contacts involving F327 (3.52 Å), Y330 (3.58 Å), W80 (3.68 Å), D68 (3.72 Å), G119 (3.75 Å), L123 (3.76 Å), and W428 (3.80 Å). The CBD–BuChE complex (Fig. 7e and S6b) was mainly stabilized by nonpolar contacts with Y438 (3.22 Å), G114 (3.25 Å), L284 (3.44 Å), E195 (3.48 Å), W80 (3.65 Å), H436 (3.71 Å), W229 (3.80 Å), F327 (3.85 Å), and Y330 (3.86 Å), consistent with its high number of BuChE contacts despite limited persistent hydrogen bonding.

In CBDA–BuChE complex (Fig. 7f and S6c), CBDA displayed hydrogen-bonding interactions with S196 (2.04 Å) and G114 (1.75 Å), G115 (2.59 Å) backbone. The complex was further stabilized by van der Waals and hydrophobic interactions with Y126 (3.29 Å), W80 (3.39–3.65 Å), F327 (3.52 Å), Y330 (3.58 Å), and W428 (3.61 Å). The Δ9-THC–BuChE complex (Fig. S4b and S6d) was dominated by hydrophobic interactions with W229 (3.38 Å), G115 (3.66 Å), W80 (3.71 Å), Y330 (3.77 Å), L284 (3.85 Å), V286 (3.85 Å), and F327 (3.86 Å). Therefore, cannabinoid binding in BuChE appears to be driven largely by hydrophobic pocket complementarity, with CBCA and CBDA gaining additional stabilization from polar contacts.

In CBCA–MAO-B complex (Fig. 7g and S7a), CBCA showed hydrogen bonding with C170 (2.20 Å) and several hydrophobic and van der Waals interactions with Q204 (3.35 Å), Y396 (3.35 Å), I197 (3.43 Å), Y324 (3.53 Å), M339 (3.57 Å), C170 (3.57 Å), and Y58 (3.63 Å). CBD showed several van der Waals and hydrophobic interactions in the binding site of MAO-B (Fig. 7h and S7b) with F341 (3.20 Å), G432 (3.46 Å), Y396 (3.52 Å), M434 (3.58 Å), Y433 (3.69 Å), Y58 (3.71 Å), I196 (3.77 Å), L169 (3.79 Å) and a hydrogen-bonding interaction with Q204 (2.28 Å), supporting its persistent association with the MAO-B cavity during MD. CBDA (Fig. 7i and S7c) showed the most favorable polar interaction profile in MAO-B, forming a hydrogen-bond with G56 – S57 (1.98 Å) backbone, together with close van der Waals and hydrophobic interactions involving Y396 (2.93 Å), Y58 (2.96 Å), G432 (3.12 Å), I196 (3.32 Å), G56 (3.42 Å), Y433 (3.47 Å), F341 (3.50 Å), and M434 (3.70 Å). The complex between Δ9-THC and MAO-B ((Fig. S3c and S7d)) was mainly stabilized by interactions with hydrophobic residues, including I197 (3.33 Å), L169 (3.34 Å), Y58 (3.40 Å), Q204 (3.58 Å), C170 (3.64 Å), I196 (3.65 Å), and P102 (3.67 Å). Δ9-THC also demonstrated a hydrogen-bonding interaction with Y324 (2.23 Å). Collectively, the combined 3D and 2D interaction analyses demonstrate that CBD, CBCA, CBDA, and Δ9-THC remained bound within the catalytic pockets of AChE, BuChE, and MAO-B after 100 ns MD simulation, with CBDA showing the clearest polar interaction contribution, CBD showing extensive contact networks, and CBCA/Δ9-THC being stabilized predominantly by hydrophobic, aromatic, and van der Waals interactions.

To further evaluate the energetic stability of the simulated complexes, MM-GBSA and MM-PBSA binding free energies were calculated using the whole 100 ns MD trajectories. All analyzed complexes showed negative binding free-energy values, indicating favorable ligand binding after MD simulation (Fig. 8). Energy component analysis (Fig. S8–S13) indicated that van der Waals interactions contributed favorably across nearly all complexes, reflecting the importance of hydrophobic packing between the cannabinoid scaffold and the enzyme binding pockets. The overall binding free-energy profiles appear to result from a balance between favorable van der Waals/electrostatic interactions and opposing polar solvation effects.

**Fig. 8:**
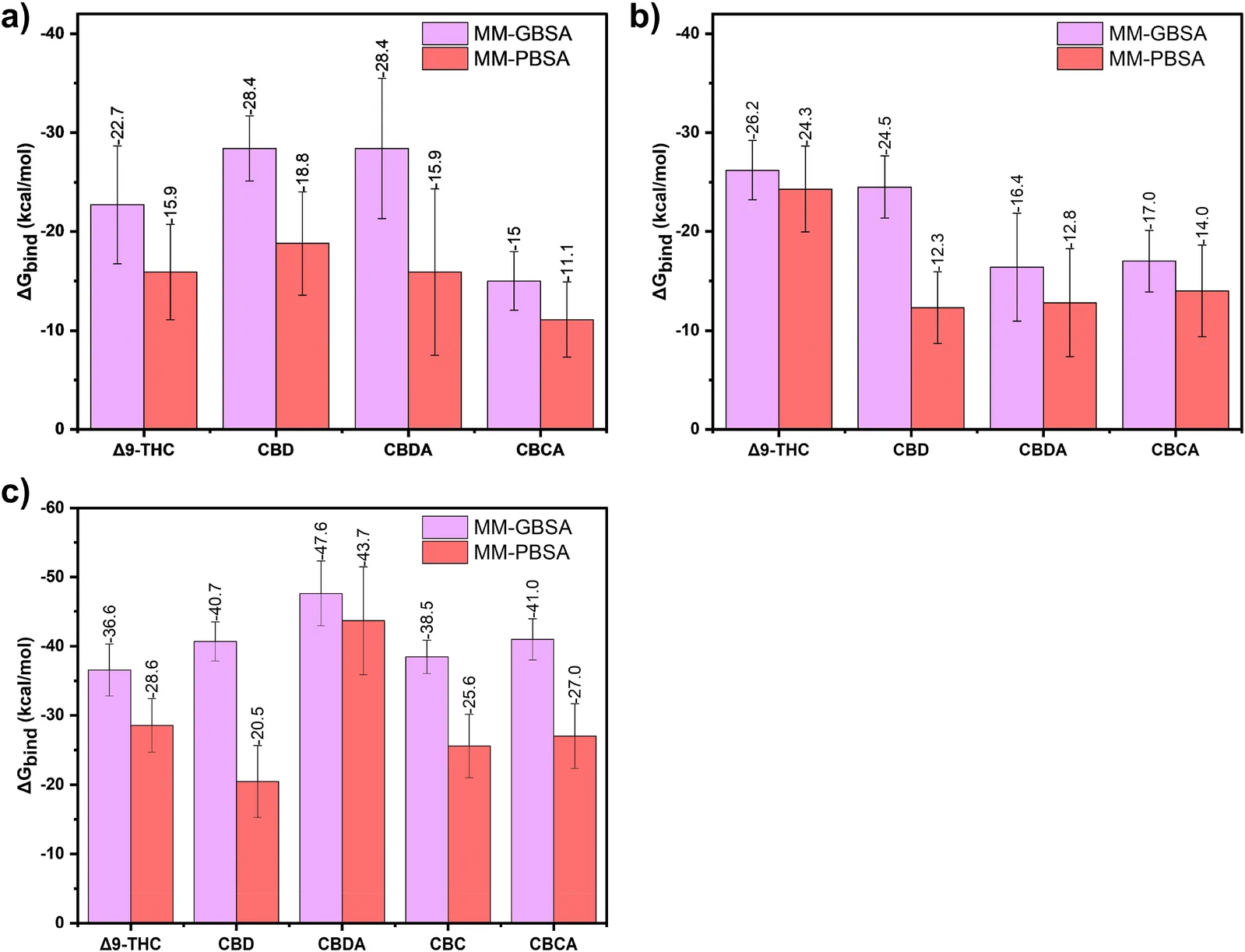
Binding free energies of ligand–enzyme complexes estimated by MM-GBSA and MM-PBSA from 100 ns MD trajectories for a) AChE, b) BuChE, and c) MAO-B.

For AChE (Figs. 8a, S8, S9), MM-GBSA identified CBDA and CBD as the most favorable ligands, with binding free energies of −28.4 ± 7.10 and −28.4 ± 3.27 kcal/mol, respectively. Δ9-THC showed an intermediate value of −22.7 ± 5.96 kcal/mol, whereas CBCA showed a weaker MM-GBSA binding energy of −15.0 ± 2.95 kcal/mol. In the MM-PBSA analysis, CBD showed the most favorable binding energy (−18.8 ± 5.23 kcal/mol), followed by Δ9-THC (−16.0 ± 4.80 kcal/mol), CBDA (−15.9 ± 8.42 kcal/mol), and CBCA (−11.1 ± 3.82 kcal/mol). These results are consistent with the MD interaction analysis, in which CBD and CBDA showed extensive contacts and shorter average minimum distances with AChE.

For BuChE (Fig. 8b–S10, S11), Δ9-THC showed the most favorable binding free energy in both MM-GBSA and MM-PBSA analyses, with values of −26.2 ± 3.02 and −24.3 ± 4.36 kcal/mol, respectively. CBD showed the second most favorable MM-GBSA value (−24.5 ± 3.15 kcal/mol), whereas CBCA and CBDA showed weaker MM-GBSA energies of −17.0 ± 3.10 and −16.4 ± 5.44 kcal/mol, respectively. In MM-PBSA, CBCA (−14.0 ± 4.62 kcal/mol), CBDA (−12.8 ± 3.15 kcal/mol), and CBD (−12.3 ± 3.62 kcal/mol) showed similar moderate binding energies. These results suggest that BuChE binding is strongly influenced by hydrophobic interactions. For MAO-B (Fig. 8c–S12, S13), CBDA showed the most favorable binding free energy by both methods, with MM-GBSA and MM-PBSA values of −47.6 ± 4.68 and −43.7 ± 7.78 kcal/mol, respectively. CBCA (−41.0 ± 2.97 kcal/mol), CBD (−40.7 ± 2.81 kcal/mol), CBC (−38.5 ± 2.40 kcal/mol), and Δ9-THC (−36.6 ± 3.74 kcal/mol) also showed favorable MM-GBSA binding energies. In MM-PBSA, CBDA (−43.7 ± 7.78 kcal/mol) remained clearly the most favorable ligand, whereas Δ9-THC (−28.6 ± 3.90 kcal/mol), CBCA (−27.0 ± 4.67 kcal/mol), CBC (−25.6 ± 4.58 kcal/mol), and CBD (−20.5 ± 5.18 kcal/mol) showed moderate binding free energies. The binding free energy values by MM-PBSA for CBCA, CBD, and Δ9-THC indicate that these ligands form more stable complexes with MAO-B than AChE and BuChE.

Overall, the *in vitro* enzyme inhibition results, molecular docking, GNINA rescoring, 100 ns MD simulations, and MM-GBSA/MM-PBSA binding free-energy analyses identify CBD, CBDA, and CBCA as the principal cannabinoid contributors to the multi-target activity of HBE. The experimental enzyme assays showed that CBCA is particularly relevant for dual cholinesterase inhibition, whereas CBD and CBDA showed stronger contributions toward MAO-B inhibition while also maintaining measurable cholinesterase activity. The computational results further supported this multi-target profile by showing that these ligands can occupy the binding pockets of AChE, BuChE, and MAO-B with stable post-MD interaction networks. Among them, CBDA showed the most favorable dynamic and energetic behavior, especially with AChE and MAO-B, supported by persistent polar interactions, shorter protein–ligand minimum distances, and favorable MM-GBSA and MM-PBSA energies. CBD maintained stable contact networks across all three enzymes, whereas CBCA retained strong binding-site interactions, particularly within the cholinesterase pockets. Although Δ9-THC showed favorable binding in some computational analyses, its weaker or absent MAO-B inhibition suggests that it is less central to the multi-target inhibitory profile than CBD, CBDA, and CBCA.

## 4 Discussion

Alzheimer's disease and related cognitive disorders involve multiple, interconnected pathological processes, including cholinergic dysfunction, monoamine oxidase-associated oxidative burden, impaired antioxidant defense, neuroinflammation, and progressive neuronal dysfunction. Therefore, agents capable of modulating more than one pathological pathway may offer advantages over single-target approaches. In the present study, HBE demonstrated a broad neuroprotective profile by inhibiting cholinesterase and MAO-B activities, reducing oxidative stress in neuronal cells, and preventing scopolamine-induced short-term cognitive dysfunction in mice. These effects were further supported by chemical profiling and computational analyses, which identified CBD, CBDA, and CBCA as major cannabinoid contributors to the multi-target activity of HBE.

Previous studies have shown that inhibition of AChE and BuChE can improve cognitive symptoms in animal models and patients with AD [61]. Current therapeutic approaches include cholinesterase inhibitors (donepezil, galantamine, and rivastigmine), the NMDA receptor antagonist (memantine), and anti-amyloid immunotherapies (lecanemab). However, despite these advances, strategies for preventing or delaying early cognitive dysfunction remain limited. Therefore, natural products capable of modulating multiple AD-related mechanisms may provide useful preventive or supportive approaches. In this context, our results identify HBE as a promising hemp-derived extract with protective effects against scopolamine-induced short-term cognitive dysfunction.

The scopolamine-induced model is widely used to evaluate AD-like cholinergic impairment and memory dysfunction [62]. Scopolamine competitively antagonizes muscarinic acetylcholine receptors, disrupts cholinergic signaling, and impairs learning and memory [63]. It can also increase AChE activity, reduce acetylcholine levels, and readily cross the blood–brain barrier. In this study, scopolamine at 1 mg/kg was administered before the passive avoidance and water maze tests, while HBE was orally administered from day 0 to day 28. The ability of HBE to improve behavioral outcomes suggests that it can protect against acute cholinergic disruption and associated memory impairment. However, because this model mainly reflects reversible cholinergic dysfunction, further studies using chronic and transgenic AD models will be required to determine whether HBE can influence long-term neurodegenerative pathology. Although the behavioral data showed consistent protective effects of HBE, the animal study was conducted with a relatively small sample size (n = 5 per group). Therefore, future studies using larger cohorts will be required to further validate the *in vivo* efficacy of HBE and its constituents.

Oxidative stress is another major contributor to neuronal damage and AD-related pathology [64], [65], [66]. Neuronal cells are particularly vulnerable to oxidative stress because of their high mitochondrial activity, oxygen demand, and lipid-rich membranes. Excessive ROS generation can damage cellular macromolecules and promote synaptic dysfunction and neuronal degeneration. Antioxidant enzymes, including SOD, catalase, and glutathione peroxidase, play essential roles in maintaining redox balance. SOD catalyzes the conversion of superoxide radicals to hydrogen peroxide, which must then be efficiently removed by downstream antioxidant systems such as catalase [67]. In our study, hemp bark and leaf extracts showed strong ABTS radical scavenging activity and significantly reduced H_2_O_2_-induced ROS production. HBE also restored SOD activity suppressed by H_2_O_2_, indicating that its neuroprotective effects involve reinforcement of antioxidant defense in addition to enzyme inhibition.

UPLC-QTOF-MS/MS profiling provided insight into the chemical basis of HBE activity. A total of 26 compounds were tentatively identified, including cannabinoids, phenolic compounds, acidic cannabinoids, and fatty acids. Among them, seven cannabinoids including CBDV, Δ9-THC, CBDA, Δ9-THCV, CBD, CBC, and CBCA were selected for enzyme inhibition assays. These cannabinoids showed distinct but complementary target profiles. CBC exhibited the strongest AChE inhibition, CBCA showed the strongest BuChE inhibition, and CBD and CBDA showed the most potent MAO-B inhibition while maintaining measurable cholinesterase activity. Thus, the biological activity of HBE is unlikely to arise from a single compound, but rather from the combined actions of multiple cannabinoids. In particular, CBCA may contribute strongly to cholinesterase inhibition, whereas CBD and CBDA may contribute more prominently to MAO-B inhibition.

Interestingly, Δ9-THC and Δ9-THCV, which are structural isomers of CBD and CBDV, respectively, did not show significant MAO-B inhibitory activity. This suggests that subtle differences in cannabinoid scaffold topology can strongly influence target selectivity. The cyclic ring system of Δ9-THC and Δ9-THCV may restrict the geometry required for productive MAO-B interactions, whereas the structures of CBD and CBDA may support more favorable binding within the MAO-B cavity. This observation indicates that the multi-target pharmacological profile of HBE depends not only on total cannabinoid content, but also on the structural diversity and target selectivity of individual cannabinoids.

The computational analyses supported the experimental enzyme inhibition results. AutoDock Vina predicted favorable binding of the tested cannabinoids to AChE, BuChE, and MAO-B, and five independent docking replicates showed consistent pose convergence. GNINA CNN-based rescoring further supported the binding-site compatibility of CBD, CBDA, and CBCA with the three enzymes. Although the computational ranking did not fully reproduce the experimental IC_50_ order for every target, this is expected because docking and rescoring do not fully account for desolvation, ligand protonation state, protein flexibility, entropy, and assay-specific enzymatic effects. Nevertheless, the combined docking and GNINA results indicate that the experimentally active cannabinoids can occupy the catalytic pockets of AChE, BuChE, and MAO-B.

The 100 ns MD simulations and post-MD interaction analyses further supported the stability of selected cannabinoid–enzyme complexes. For AChE, CBDA and CBD showed stable interaction profiles with extensive contacts, short minimum distances, and persistent hydrogen-bonding behavior. For BuChE, CBD maintained the highest number of protein–ligand contacts, whereas CBCA and CBDA gained additional stabilization through polar interactions. For MAO-B, CBD and CBDA showed the most stable RMSD profiles, and CBDA displayed the strongest hydrogen-bonding pattern and shortest average minimum distance. The 3D and 2D interaction maps confirmed that these ligands remained within the enzyme binding pockets and interacted with key residues through hydrophobic, van der Waals, aromatic, and polar contacts. These results support CBD, CBDA, and CBCA as major HBE-derived contributors to multi-target enzyme modulation.

MM-GBSA and MM-PBSA analyses further confirmed the energetic stability of the simulated complexes. For AChE, CBD and CBDA showed the most favorable binding free-energy profiles, consistent with their stable MD contacts. For BuChE, Δ9-THC and CBD showed favorable binding energies, suggesting an important role of hydrophobic interactions within the BuChE binding site. For MAO-B, CBDA showed the most favorable binding free energy by both MM-GBSA and MM-PBSA, followed by CBCA and CBD. Energy component analysis indicated that van der Waals interactions contributed favorably across nearly all complexes, consistent with hydrophobic packing between cannabinoid scaffolds and enzyme binding pockets. These computational results reinforce the experimental finding that HBE contains multiple cannabinoids capable of modulating enzymes associated with cognitive dysfunction.

There are a few limitations in this study that should be taken in consideration. The animal experiments were conducted with a relatively small sample size and only male mice. Thus, future studies should include larger cohorts and both sexes. In addition, the scopolamine model is useful for studying acute cholinergic dysfunction, but it does not fully reproduce progressive AD pathology. Further studies using chemically induced models, such as streptozotocin or colchicine-induced models, and transgenic models such as 3xTg or 5xFAD mice will be necessary to evaluate the long-term preventive and therapeutic potential of HBE. Finally, although CBD, CBDA, and CBCA were identified as important contributors, HBE is chemically complex, and other constituents may also participate in its antioxidant and neuroprotective effects. Direct target-engagement studies will also be required to validate the predicted binding modes experimentally.

Despite these limitations, the present findings identify HBE as a chemically rich hemp-derived extract with multi-target activity relevant to cognitive dysfunction. HBE inhibited cholinesterase and MAO-B activity, reduced oxidative stress, restored antioxidant defense, and prevented scopolamine-induced behavioral deficits. The combined experimental and computational analyses further identified CBD, CBDA, and CBCA as major contributors to this multi-target profile. Further optimization of HBE extraction, standardization of active cannabinoid content, long-term safety evaluation, and validation in chronic cognitive impairment models may support the development of HBE as a preventive or supportive natural-product strategy for AD-related cognitive dysfunction.

## 5 Conclusion

In this study, HBE demonstrated significant multi-target activity relevant to AD-related cognitive dysfunction. Among the ethanol extracts prepared from different anatomical parts of hemp, HBE showed a balanced pharmacological profile by inhibiting AChE, BuChE, and MAO-B activities, reducing oxidative stress, and restoring antioxidant defense in SH-SY5Y neuronal cells. In the scopolamine-induced mouse model, oral administration of HBE prevented short-term cognitive impairment, as shown by improved performance in the passive avoidance and water maze tests. These results suggest that HBE can protect against acute cholinergic dysfunction and oxidative stress-associated memory impairment. The UPLC-QTOF-MS/MS analysis revealed that HBE contains chemically diverse constituents, including multiple cannabinoids. Among the identified cannabinoids, CBD, CBDA, and CBCA showed the most relevant multi-target profiles. CBCA contributed strongly to cholinesterase inhibition, whereas CBD and CBDA showed stronger MAO-B inhibitory activity while retaining measurable cholinesterase inhibition. The *in silico* analyses further supported the ability of these cannabinoids to bind stably within the active-site regions of AChE, BuChE, and MAO-B. In particular, CBDA showed favorable dynamic and energetic behavior with AChE and MAO-B, while CBD maintained stable interaction networks across all three enzymes. Overall, the findings of this study indicate that the protective effects of HBE are likely mediated by the complementary actions of multiple cannabinoids rather than a single active constituent. HBE may therefore serve as a promising natural-product source for the development of preventive or supportive strategies against cognitive dysfunction associated with cholinergic deficit, MAO-B activity, and oxidative stress. Further studies using chronic and transgenic models of neurodegeneration, along with extract standardization and long-term safety evaluation, are warranted to validate its therapeutic potential.

## CRediT authorship contribution statement

Hee-Young Heo: Investigation, Methodology, Formal analysis, Data curation, Jae Kwon Kim: Writing – original draft, Methodology, Formal analysis, Data curation, SeonJu Park: Investigation, Validation, Formal analysis, Keum-Soo Song: Methodology, Data curation, Satish Balasaheb Nimse: Writing – original draft, Software, Methodology, Formal analysis, Data curation, Resources, Project administration, Kooyeon Lee: Conceptualization, Supervision, Funding acquisition, Project administration, Writing – review & editing.

## Ethics declaration

This study was conducted in accordance with the following guidelines for animal welfare and/or reporting: Institutional Animal Care and Use Committee of the Southeast Medi-Chem Institute. This study was approved by the Institutional Animal Care and Use Committee of the Southeast Medi-Chem Institute. (Approval No. Approval No. SEMI-20-014)

## Declaration of competing interest

The authors declare that they have no known competing financial interests or personal relationships that could have appeared to influence the work reported in this paper.

## Data Availability

The complete *in silico* dataset supporting this study including molecular docking and GNINA rescoring inputs and results, 100 ns molecular dynamics analysis outputs, MM-GBSA/MM-PBSA binding free-energy data, interaction maps, and all analysis scripts is openly available in Zenodo at https://doi.org/10.5281/zenodo.21712130. All other data is available upon request. Supplementary material is in the online version.
