# Supplementary material for: Hemp bark extract attenuates scopolamine-induced cognitive dysfunction through multi-target cholinesterase, MAO-B, and antioxidant mechanisms
Source: Biochem Biophys Rep. 2026 Oct 28;48:102852. doi: 10.1016/j.bbrep.2026.102852 (PMC13742105; doi:10.1016/j.bbrep.2026.102852)
Supplement: Supplementary file 1 [file mmc1.docx]

**Hemp bark extract attenuates scopolamine-induced cognitive dysfunction through multi-target cholinesterase, MAO-B, and antioxidant mechanisms**

Hee-Young Heo^a^, Jae Kwon Kim^a^, SeonJu Park^b^ Keum-Soo Song^a^, Satish Balasaheb Nimse*^, c,e,f^, Kooyeon Lee*^, a,d^

^a^Research Institute, K-medichem Co., Ltd., Chuncheon, 24341, Republic of Korea

^b^Metropolitan Seoul Center, Korea Basic Science Institute, Seoul, Republic of Korea

^c^OBMC Lab, Major in Smart-Healthcare Convergence, Hallym University, Chuncheon, 24252, Republic of Korea

^d^Department of Bio-Health Technology, College of Biomedical Science, Kangwon National University, Chuncheon, 24341, Republic of Korea

^e^Multidisciplinary Genome Institute, Hallym University, Chuncheon 24252, Republic of Korea.

^f^Hallym AI-Biohealth R&BD center, Hallym University, Chuncheon 24252, Republic of Korea.

Corresponding Authors:

Satish Balasaheb Nimse (satish_nimse@hallym.ac.kr), Kooyeon Lee (lky@kangwon.ac.kr)

**Table S1. Constituents identified in hemp bark extract using UPLC-QTOF-MS**

| **No** | **Retention Time (min)** | **m/z**  **[M-H]-** | **Mass error (ppm)** | **Response** | **MS^2^(m/z)** | **Tentative identification** |
| --- | --- | --- | --- | --- | --- | --- |
| 1 | 0.73 | 341.1094 | 1.4 | 4063551 | 179.0564 | β-Gentiobiose |
| 2 | 0.77 | 359.0980 | -0.9 | 3764 | 161.0458, 179.0564, 197.0457 | Syringic acid-O-hexoside |
| 3 | 1.03 | 356.2445 | 0.6 | 15514 | - | C19H35NO5 |
| 4 | 1.06 | 293.1767 | 2.9 | 391150 | - | C17H26O4 |
| 5 | 1.14 | 360.2756 | 0.3 | 21487 | - | C19H39NO5 |
| 6 | 1.29 | 353.1746 | -3.5 | 2941 | 279.1400, 309.1855 | Cannabinodiolic acid (CBNDA) |
| **7** | **1.53** | **285.1854** | **-1.9** | **7228** | **151.0760, 163.0760, 217.1227** | **Cannabidivarin (CBDV)** |
| 8 | 1.53 | 329.1750 | -2.5 | 468524 | 217.1227, 311.16  50 | Cannabidivarinic acid (CBDVA) |
| 9 | 1.61 | 277.2169 | -1.5 | 3041 | - | gamma-linolenic acid |
| **10** | **2.01** | **313.2177** | **1.1** | **97319** | **179.1079, 245.1547** | **Δ9-Tetrahydrocannabinol (Δ9-THC)** |
| **11** | **2.01** | **357.2074** | **0.7** | **2230389** | **107.0505, 137.0974, 245.1551, 311.2021, 339.1971** | **Cannabidiolic acid (CBDA)** |
| 12 | 2.02 | [M+COOH]-  389.1971 | 0.3 | 370406 | 245.1551 | 8,11-Bis-OH-9-THCA |
| 13 | 2.26 | 656.2535 | 2.3 | 236302 | - | C25H43N3O17 |
| 14 | 2.83 | 329.2138 | 4.7 | 7163 | 179.1087, 271.1715, 311.2005 | Cannabielsoin (CBE) |
| **15** | **3.22** | **285.1868** | **2.6** | **12239** | **163.0769, 217.1240** | **Δ9-Tetrahydrocannabivarin (Δ9-THCV)** |
| 16 | 3.22 | 329.1767 | 2.5 | 453534 | 163.0769, 217.1240, 285.1870, 311.1670 | Tetrahydrocannabivarinic acid (THCVA) |
| 17 | 3.52 | 265.1486 | -1.6 | 157708 | - | C5H18N10O3 |
| 18 | 3.56 | 277.2181 | 3 | 3869 | - | alpha-linolenic acid |
| 19 | 3.95 | 225.2222 | -0.2 | 28248 | - | n-Pentadecanal |
| 20 | 4.30 | 353.1758 | 0 | 319763 | 279.1391, 309.1859 | Cannabinolic acid (CBNA) |
| 21 | 5.18 | 279.2325 | -1.4 | 8197 | - | linoleic acid |
| **22** | **5.42** | **313.2167** | **-2.1** | **104914** | **179.1073, 191.1074, 245.1543** | **Cannabidiol (CBD)** |
| 23 | 5.43 | 357.2067 | -1.1 | 2053468 | 191.1074, 245.1543, 313.2169 | Δ9-tetrahydrocannabinolic acid A (THCAA) |
| **24** | **6.32** | **313.2178** | **1.5** | **3537** | **179.1080, 191.1080, 243.1394** | **Cannabichromene (CBC)** |
| **25** | **6.32** | **357.2075** | **0.9** | **119673** | **191.1080, 313.2181** | **Cannabichromenic acid (CBCA)** |
| 26 | 7.20 | 255.2332 | 1.0 | 9093 | - | Hexadecanoic acid |

**Figure S1.** Chemical structures of representative constituents identified in HBE using UPLC-QTOF-MS

**Table S2**. Binding site coordinates and 3D grid box dimensions used in the docking study of seven cannabinoids with AChE, BuChE, and MAO-B.

| **Protein** | **PDB ID** | **Binding site coordinates (Å)** | | | **3D gid box (Å)** | | |
| --- | --- | --- | --- | --- | --- | --- | --- |
|  |  | center_x | center_y | center_z | size_x | size_y | size_z |
| AChE | 4EY7 | -14.4343 | -42.9858 | 28.153 | 25.5963 | 21.9355 | 25.1356 |
| BuChE | 5DYY | 3.9259 | -9.43877 | -13.0461 | 17.5889 | 22.0908 | 24.003 |
| MAO-B | 2V5Z | 52.3715 | 156.191 | 28.7744 | 16.1861 | 16.4929 | 16.5168 |

**Table S3.** The RMSD (mean ± SD) values and consensus (n/5, 2.0 Å cut-off) for the top-ranked binding poses of seven cannabinoids docked (n = 5) to AChE, BuChE, and MAO-B enzymes.

| **Ligand** | **AChE** | | **BuChE** | | **MAO-B** | |
| --- | --- | --- | --- | --- | --- | --- |
|  | **RMSD**  **(Å)** | **Consensus**  **(n/5)** | **RMSD**  **(Å)** | **Consensus**  **(n/5)** | **RMSD**  **(Å)** | **Consensus**  **(n/5)** |
| **E20** | 0.02 ± 0.01 | - | - | - | - | - |
| **5HH** | - | - | 0.05 ± 0.03 | - | - | - |
| **SAG** | - | - | - | - | 0.54 ± 0.71 | - |
| **CBDV** | 0.01 ± 0.01 | 5/5 | 0.01 ± 0.01 | 5/5 | 0.01 ± 0.01 | 5/5 |
| **Δ9-THC** | 0.02 ± 0.01 | 5/5 | 0.04 ± 0.02 | 5/5 | 0.01 ± 0.01 | 5/5 |
| **CBDA** | 1.61 ± 1.21 | 5/5 | 0.09 ± 0.06 | 5/5 | 0.36 ± 0.61 | 5/5 |
| **Δ9-THCV** | 0.01 ± 0.01 | 5/5 | 0.03 ± 0.02 | 5/5 | 0.01 ± 0.01 | 5/5 |
| **CBD** | 0.03 ± 0.02 | 5/5 | 0.04 ± 0.04 | 5/5 | 0.37 ± 0.77 | 5/5 |
| **CBC** | 0.05 ± 0.04 | 5/5 | 0.58 ± 0.68 | 5/5 | 0.04 ± 0.02 | 5/5 |
| **CBCA** | 0.19 ± 0.29 | 5/5 | 0.64 ± 0.82 | 5/5 | 0.05 ± 0.04 | 5/5 |

E20, 5HH, and SAG are the co-crystalized ligands in AChE, BuChE, and MAO-B, respectively. The RMSD values for these ligands were calculated against the crystallographic native-ligand pose.

**Table S4.** GNINA CNN affinity (*K_d_*, Mean ± S.D.) and CNN score (probability, Mean ± S.D.) for binding interactions of phytochemicals docked (n = 5) to various enzymes.

| **Ligand** | **AChE** | | **BuChE** | | **MAO-B** | |
| --- | --- | --- | --- | --- | --- | --- |
|  | **CNN**  **affinity** | **CNN**  **score** | **CNN**  **affinity** | **CNN**  **score** | **CNN**  **affinity** | **CNN**  **score** |
| **CBDV** | 5.91 ± 0.04 | 0.29 ± 0.00 | 6.50 ± 0.01 | 0.83 ± 0.01 | 4.77 ± 0.03 | 0.12 ± 0.00 |
| **Δ9-THC** | 6.52 ± 0.03 | 0.70 ± 0.01 | 5.74 ± 0.02 | 0.28 ± 0.01 | 7.06 ± 0.01 | 0.86 ± 0.00 |
| **CBDA** | 6.25 ± 0.03 | 0.66 ± 0.03 | 7.04 ± 0.04 | 0.90 ± 0.01 | 5.61 ± 0.59 | 0.17 ± 0.07 |
| **Δ9-THCV** | 6.00 ± 0.01 | 0.60 ± 0.01 | 6.29 ± 0.03 | 0.40 ± 0.02 | 5.05 ± 0.01 | 0.13 ± 0.00 |
| **CBD** | 6.74 ± 0.01 | 0.42 ± 0.01 | 6.93 ± 0.01 | 0.95 ± 0.00 | 5.17 ± 0.02 | 0.09 ± 0.00 |
| **CBC** | 6.27 ± 0.01 | 0.35 ± 0.00 | 6.66 ± 0.07 | 0.80 ± 0.09 | 5.68 ± 0.03 | 0.20 ± 0.00 |
| **CBCA** | 6.19 ± 0.03 | 0.30 ± 0.01 | 6.87 ± 0.17 | 0.82 ± 0.20 | 4.95 ± 0.03 | 0.14 ± 0.00 |

**Table S5.** Summary of MD simulation analyses for selected enzyme–cannabinoid complexes.

| **Target** | **Ligand** | **Protein RMSD**  **(nm)**  **mean ± SD** | **Protein RMSF**  **(nm)**  **mean ± SD** | **Rg**  **(nm)**  **mean ± SD** | **H-bonds (n)**  **mean ± SD** | **Contacts (n)**  **<3.5 (Å)**  **mean ± SD** | **MD**  **(Å)**  **mean ± SD** |
| --- | --- | --- | --- | --- | --- | --- | --- |
| **AChE** | CBCA | 0.170 ± 0.01 | 0.118 ± 0.07 | 2.32 ± 0.01 | - | 39.3 ± 4.71 | 2.06 ± 0.11 |
|  | CBD | 0.195 ± 0.01 | 0.115 ± 0.09 | 2.32 ± 0.01 | 0.97 ± 0.35 | 47.4 ± 1.97 | 1.89 ± 0.11 |
|  | CBDA | 0.180 ± 0.01 | 0.082 ± 0.05 | 2.32 ± 0.01 | 3.41 ± 1.23 | 47.7 ± 3.25 | 1.75 ± 0.11 |
|  | Δ9-THC | 0.201 ± 0.01 | 0.124 ± 0.08 | 2.32 ± 0.01 | 0.74 ± 0.50 | 42.2 ± 3.39 | 1.96 ± 0.12 |
| **BuChE** | CBCA | 0.194 ± 0.01 | 0.129 ± 0.08 | 2.35 ± 0.01 | 0.13 ± 0.35 | 38.8 ± 4.24 | 2.02 ± 0.14 |
|  | CBD | 0.156 ± 0.01 | 0.122 ± 0.07 | 2.32 ± 0.01 | - | 44.5 ± 3.35 | 2.02 ± 0.11 |
|  | CBDA | 0.193 ± 0.01 | 0.130 ± 0.08 | 2.34 ± 0.01 | 0.01 ± 0.08 | 31.1 ± 7.33 | 2.12 ± 0.14 |
|  | Δ9-THC | 0.168 ± 0.01 | 0.122 ± 0.07 | 2.34 ± 0.01 | 0.17 ± 0.38 | 41.6 ± 3.04 | 1.10 ± 0.14 |
| **MAO-B** | CBC | 0.318 ± 0.05 | 0.152 ± 0.12 | 2.38 ± 0.01 | 0.96 ± 0.23 | 47.6 ± 2.51 | 1.82 ± 0.13 |
|  | CBCA | 0.362 ± 0.02 | 0.147 ± 0.13 | 2.36 ± 0.01 | 0.34 ± 0.49 | 50.2 ± 2.60 | 1.93 ± 0.13 |
|  | CBD | 0.168 ± 0.02 | 0.130 ± 0.08 | 2.38 ± 0.01 | 2.23 ± 0.55 | 50.2 ± 1.52 | 1.83 ± 0.11 |
|  | CBDA | 0.148 ± 0.02 | 0.128 ± 0.08 | 2.39 ± 0.01 | 3.07 ± 0.43 | 48.6 ± 2.42 | 1.64 ± 0.09 |
|  | Δ9-THC | 0.388 ± 0.01 | 0.147 ± 0.15 | 2.36 ± 0.01 | 0.41 ± 0.54 | 48.8 ± 2.14 | 1.95 ± 0.10 |

**Figure S2**. Plots, a) Root-mean-square deviation (RMSD), b) Root-mean-square fluctuation (RMSF), c) Number of protein-ligand contacts (< 3.5 Å), d) Radius of gyration (Rg), e) Number of hydrogen-bond interactions, and f) Protein-ligand average minimum distances (Å), representing results of 100 ns MD simulation for BuChE complexes with CBCA, CBD, CBDA, and Δ9-THC.

**Figure S3**. Plots, a) Root-mean-square deviation (RMSD), b) Root-mean-square fluctuation (RMSF), c) Number of protein-ligand contacts (< 3.5 Å), d) Radius of gyration (Rg), e) Number of hydrogen-bond interactions, and f) Protein-ligand average minimum distances (Å), representing results of 100 ns MD simulation for MAO-B complexes with CBC, CBCA, CBD, CBDA, and Δ9-THC.

**Figure S4.** Ligand–protein interactions analyzed for Δ9-THC with enzymes a) AChE, b) BuChE, c) MAO-B upon 100 ns MD simulation (interacting amino acids; blue, Δ9-THC; orange, red dotted lines, hydrogen-bonding interactions).

**Figure S5.** Ligand–protein interactions analyzed for a) CBCA, b) CBD, c) CBDA, and d) Δ9-THC with AChE upon 100 ns MD simulation (The 2D interaction maps were generated using ProLIF).

**Figure S6.** Ligand–protein interactions analyzed for a) CBCA, b) CBD, c) CBDA, and d) Δ9-THC with BuChE upon 100 ns MD simulation (The 2D interaction maps were generated using ProLIF).

**Figure S7.** Ligand–protein interactions analyzed for a) CBCA, b) CBD, c) CBDA, and d) Δ9-THC with MAO-B upon 100 ns MD simulation (The 2D interaction maps were generated using ProLIF).

**Figure S8.** Energy component analysis for relative binding free energy calculation by MM-GBSA for the interactions of ligands and AChE.

**Figure S9.** Energy component analysis for relative binding free energy calculation by MM-PBSA for the interactions of ligands and AChE.

**Figure S10.** Energy component analysis for relative binding free energy calculation by MM-GBSA for the interactions of ligands and BuChE.

**Figure S11.** Energy component analysis for relative binding free energy calculation by MM-PBSA for the interactions of ligands and BuChE.

**Figure S12.** Energy component analysis for relative binding free energy calculation by MM-GBSA for the interactions of ligands and MAO-B.

**Figure S13.** Energy component analysis for relative binding free energy calculation by MM-PBSA for the interactions of ligands and MAO-B.
